# Barcoded oligonucleotides ligated on RNA amplified for multiplexed and parallel *in situ* analyses

**DOI:** 10.1093/nar/gkab120

**Published:** 2021-03-08

**Authors:** Songlei Liu, Sukanya Punthambaker, Eswar P R Iyer, Thomas Ferrante, Daniel Goodwin, Daniel Fürth, Andrew C Pawlowski, Kunal Jindal, Jenny M Tam, Lauren Mifflin, Shahar Alon, Anubhav Sinha, Asmamaw T Wassie, Fei Chen, Anne Cheng, Valerie Willocq, Katharina Meyer, King-Hwa Ling, Conor K Camplisson, Richie E Kohman, John Aach, Je Hyuk Lee, Bruce A Yankner, Edward S Boyden, George M Church

**Affiliations:** Department of Genetics, Harvard Medical School, Boston, MA 02115, USA; Department of Genetics, Harvard Medical School, Boston, MA 02115, USA; Wyss Institute for Biologically Inspired Engineering, Harvard University, Boston, MA 02115, USA; Department of Genetics, Harvard Medical School, Boston, MA 02115, USA; Wyss Institute for Biologically Inspired Engineering, Harvard University, Boston, MA 02115, USA; Wyss Institute for Biologically Inspired Engineering, Harvard University, Boston, MA 02115, USA; McGovern Institute, Massachusetts Institute of Technology, Cambridge, MA 02139, USA; Media Arts and Sciences, Massachusetts Institute of Technology, Cambridge, MA 02139, USA; Cold Spring Harbor Laboratory, Cold Spring Harbor, NY 11724, USA; Department of Genetics, Harvard Medical School, Boston, MA 02115, USA; Wyss Institute for Biologically Inspired Engineering, Harvard University, Boston, MA 02115, USA; Department of Genetics, Harvard Medical School, Boston, MA 02115, USA; Wyss Institute for Biologically Inspired Engineering, Harvard University, Boston, MA 02115, USA; Department of Genetics, Harvard Medical School, Boston, MA 02115, USA; Wyss Institute for Biologically Inspired Engineering, Harvard University, Boston, MA 02115, USA; Department of Genetics, Harvard Medical School, Boston, MA 02115, USA; McGovern Institute, Massachusetts Institute of Technology, Cambridge, MA 02139, USA; Media Arts and Sciences, Massachusetts Institute of Technology, Cambridge, MA 02139, USA; McGovern Institute, Massachusetts Institute of Technology, Cambridge, MA 02139, USA; Media Arts and Sciences, Massachusetts Institute of Technology, Cambridge, MA 02139, USA; Harvard-MIT Health Sciences and Technology, Massachusetts Institute of Technology, Cambridge, MA 02139, USA; McGovern Institute, Massachusetts Institute of Technology, Cambridge, MA 02139, USA; Media Arts and Sciences, Massachusetts Institute of Technology, Cambridge, MA 02139, USA; Department of Biological Engineering, Massachusetts Institute of Technology, Cambridge, MA 02142, USA; Media Arts and Sciences, Massachusetts Institute of Technology, Cambridge, MA 02139, USA; Broad Institute, Massachusetts Institute of Technology, Cambridge, MA 02142, USA; Wyss Institute for Biologically Inspired Engineering, Harvard University, Boston, MA 02115, USA; Wyss Institute for Biologically Inspired Engineering, Harvard University, Boston, MA 02115, USA; Department of Genetics, Harvard Medical School, Boston, MA 02115, USA; Department of Genetics, Harvard Medical School, Boston, MA 02115, USA; Department of Biomedical Sciences, Faculty of Medicine and Health Sciences, Universiti Putra Malaysia, 43400 Serdang, Selangor, Malaysia; Department of Genetics, Harvard Medical School, Boston, MA 02115, USA; Wyss Institute for Biologically Inspired Engineering, Harvard University, Boston, MA 02115, USA; Department of Genetics, Harvard Medical School, Boston, MA 02115, USA; Wyss Institute for Biologically Inspired Engineering, Harvard University, Boston, MA 02115, USA; Department of Genetics, Harvard Medical School, Boston, MA 02115, USA; Cold Spring Harbor Laboratory, Cold Spring Harbor, NY 11724, USA; Department of Genetics, Harvard Medical School, Boston, MA 02115, USA; McGovern Institute, Massachusetts Institute of Technology, Cambridge, MA 02139, USA; Media Arts and Sciences, Massachusetts Institute of Technology, Cambridge, MA 02139, USA; Department of Biological Engineering, Massachusetts Institute of Technology, Cambridge, MA 02142, USA; Koch Institute for Integrative Cancer Research, Massachusetts Institute of Technology, Cambridge, MA 02142, USA; Department of Brain and Cognitive Sciences, Massachusetts Institute of Technology, Cambridge, MA 02139, USAHoward Hughes Medical Institute, Chevy Chase, MD 20815, USA; Department of Genetics, Harvard Medical School, Boston, MA 02115, USA; Wyss Institute for Biologically Inspired Engineering, Harvard University, Boston, MA 02115, USA

## Abstract

We present **b**arcoded **o**ligonucleotides **l**igated **o**n **R**NA **a**mplified for **m**ultiplexed and parallel ***i****n****s****itu* analyses (BOLORAMIS), a reverse transcription-free method for spatially-resolved, targeted, *in situ* RNA identification of single or multiple targets. BOLORAMIS was demonstrated on a range of cell types and human cerebral organoids. Singleplex experiments to detect coding and non-coding RNAs in human iPSCs showed a stem-cell signature pattern. Specificity of BOLORAMIS was found to be 92% as illustrated by a clear distinction between human and mouse housekeeping genes in a co-culture system, as well as by recapitulation of subcellular localization of lncRNA *MALAT1*. Sensitivity of BOLORAMIS was quantified by comparing with single molecule FISH experiments and found to be 11%, 12% and 35% for *GAPDH*, *TFRC* and *POLR2A*, respectively. To demonstrate BOLORAMIS for multiplexed gene analysis, we targeted 96 mRNAs within a co-culture of iNGN neurons and HMC3 human microglial cells. We used fluorescence *in situ* sequencing to detect error-robust 8-base barcodes associated with each of these genes. We then used this data to uncover the spatial relationship among cells and transcripts by performing single-cell clustering and gene–gene proximity analyses. We anticipate the BOLORAMIS technology for *in situ* RNA detection to find applications in basic and translational research.

## INTRODUCTION

Spatial transcriptomics is a rapidly evolving field, with recent developments in multiplexed *in situ* technologies paving the way for spatial imaging of the genome and transcriptome at an unprecedented resolution (1). Spatial profiling of gene expression patterns in cells of a given tissue can provide intricate molecular maps, thus allowing quantification of transcripts and understanding of cellular function in a particular environment. We proposed the idea of *in situ* DNA amplification using PCR in 1999 (2) and advanced this method to a technology called Fluorescent *In Situ* Sequencing (FISSEQ) in 2003 that used fluorescent deoxynucleotides for sequencing polonies (3,4). FISSEQ enables high-throughput multiplexing along with high resolution imaging of targets, while keeping morphology of the samples intact, thus preserving the cellular context, as compared with bulk sequencing methods. Although our idea of performing FISSEQ in cells was intrinsic to its conception, it was from 2013 onwards that the generation and sequencing of highly multiplexed and spatially resolved *in situ* RNA libraries in cells and tissues was demonstrated (5,6). While FISSEQ offers *de novo* transcriptome sequencing and therefore is not targeted to specific transcripts, the detection efficiency and sensitivity is low (<0.005%) (7).

Targeted *in situ* detection of messenger RNA (mRNA) gives higher efficiency by reducing background (by relieving crowding of targets and competition for reagents compared to whole-transcriptome), thereby increasing the signal-to-noise ratio. This can be achieved by using barcoded padlock probes designed for specific targets, amplifying the circularized probes and sequencing the barcodes over multiple rounds. This method provides a means to map the expression profile of a subset of transcripts and compare it to the expression levels in surrounding cells in a given tissue.

Padlock probes have been demonstrated for *in situ* sequencing for multiplexed transcriptomics, with single-base resolution on a small number of transcripts (6). However, in both this method and FISSEQ, detection efficiency is a function of the reverse transcription (RT) step, that results in a DNA–RNA hybrid, after the formation of which the RNA needs to be digested away to expose the cDNA. Thus, this RT step is subject to noise resulting from variable priming efficiency and random priming induced bias (8). Locked nucleic acid modified primers have been demonstrated to increase RT efficiency with padlock probes, but require careful calibration and can be cost-prohibitive for genome-wide applications (5,6,9).

Previously described spatial transcriptomic technologies that do not require reverse transcription include MERFISH (10,11), SeqFISH+ (12,13) and STARmap (14). Both MERFISH and SeqFISH+ require the targets to be at least around 1kb to produce enough space for encoding probes to hybridize, limiting the detection of short transcripts. STARmap devised a smart strategy of direct short-footprint RNA targeting using SNAIL (specific amplification of nucleic acids via intramolecular ligation) probes, where two pieces of DNA probes act together to create a circular template upon co-hybridization to the same RNA molecule. The template length required by SNAIL probes is 40–46 nucleotides (nt). Although much shorter than MERFISH and SeqFISH+, SNAIL probes used by STARmap do not have the potential to distinguish finer distinctions between transcripts like point mutations or targeting even shorter transcripts like microRNAs (miRNAs).

**Figure 1.**
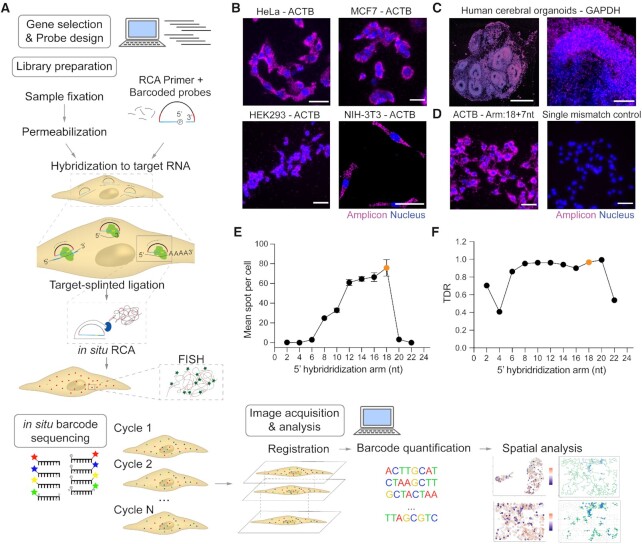
BOLORAMIS workflow and probe optimization. (**A**) Schematic of BOLORAMIS workflow. Colors in barcoded probes: blue – hybridization arm, red – barcodes, black – RCA priming region. Example images of BOLORAMIS library preparation on (**B**) HeLa (scale bar: 50 μm), HEK293, MCF7, NIH-3T3 cells and (**C**) human cerebral organoids (scale bar: left 500 μm, right 100 μm). (**D**) Representative image of *ACTB* probe with 18 nt 5′ arm and 7 nt 3′ arm; and its ligation junction single mismatch control (scale bar: 50 μm). (**E**) Mean spot per cell and (**F**) true discovery rate (TDR, see Materials and Methods) for *ACTB* in HeLa cells with the 5′ hybridization arm from 2 nt to 22 nt. Orange dots are 18 nt.

To overcome these issues related to the RT step and requirements for long target sequences, we developed BOLORAMIS (barcoded oligonucleotides ligated on RNA amplified for multiplexed and parallel *insitu* analyses), a reverse transcription-free direct RNA detection method. In this study, we have optimized probe design, demonstrated specificity and sensitivity, as well as multiplexing capabilities of the method on a set of 96 genes. BOLORAMIS is based on combinatorial molecular indexing combined with direct RNA-dependent ligation and clonal amplification of barcoded padlock probes. In this method, after cell fixation and permeabilization, the probes are added so that they directly hybridize to RNA molecules – thus eliminating the need for conversion of the RNA to cDNA by RT. The probes are then ligated using SplintR Ligase (15) and subjected to rolling circle amplification (RCA) to produce amplicons. The amplicons are then crosslinked to the cellular matrix to prevent translocation and the barcodes are detected by fluorescent *in situ* hybridization (FISH) or sequenced *in situ* to get a high-resolution imaging readout of the bases they encode, to thereby reveal probe identity (Figure 1A). We demonstrate that BOLORAMIS works on diverse cell and tissue types including human cerebral organoids. Using our BOLORAMIS method, we were able to map the spatial patterns of cells and genes by targeting 96 mRNAs within a co-culture of iNGN neurons (16) and HMC3 human microglial cells (17).

## MATERIALS AND METHODS

### Cell culture

Regular maintenance and passaging of cells were performed in standard tissue culture plates. HeLa, MCF7, NIH-3T3 and HMC3 (ATCC, CRL-3304) cells were cultured in DMEM/F12 (with 10% Fetal Bovine Serum (FBS)) changed every 48 h. Uninduced PGP1 iNGN cells were cultured as described earlier (16). Briefly, tissue culture plates were precoated with Matrigel (Corning, 354277) following manufacturer's instructions. Induced pluripotent stem cells (iPSCs) were maintained in mTeSR1 (Stemcell Technologies, 05850) changed every 24 h. For passaging, cells were incubated with TrypLE Express Enzyme (Thermo Fisher Scientific, 12604013) for 1–5 min in the incubator until cells started to detach. 1.5× volume of culture media were added to terminate the digestion. After gentle aspiration, the entire volume was collected and centrifuged at 300 g for 5 min. Supernatant was removed and cells were resuspended in culture media. Cells were counted and re-plated to reach 80% confluency after 4 days. iPSCs were re-plated in fresh mTeSR1 media containing 10 μM Y-27632 (ROCK inhibitor) (Stemcell Technologies, 72302). For BOLORAMIS library preparation and imaging, all cell lines used in this paper were plated on removable chambered coverglass (Grace Bio-Labs, 112358). For the co-culture of iNGN with HMC3, monoculture of iNGN iPSCs were induced with 0.5 μg/ml doxycycline (Sigma, D3072) after passaging for 4 days. On day 3, monoculture of HMC3 were dissociated and added to the culture of iNGN for 24 h prior to fixation.

### BOLORAMIS library preparation for non-384 well experiments

Cells were fixed with 4% paraformaldehyde (Electron Microscopy Sciences) for 15 min, rinsed with phosphate-buffered saline (PBS) (Life Technologies) and permeabilized with 0.25% Triton X-100 (Thermo Fisher Scientific) for 15 min. Padlock probes (1 μM final probe concentration for singleplex experiments, 10 μM for multiplexed experiments) along with RCA primer (1:1 mole ratio) in Hybridization Buffer (10% formamide in 6× Saline Sodium Citrate (SSC) buffer) were added to the sample and incubated for 1 h at 37°C, followed by three 5-min washes with Hybridization Buffer to remove excess unhybridized primer and probe, followed by a wash with 1× SplintR Ligase buffer. Ligase mix containing 210 nM (1:50) SplintR Ligase (New England Biolabs) in 1× SplintR Ligase Reaction Buffer was added to the sample and incubated for 1 h at room temperature followed by three rinses with the Hybridization Buffer to remove the unligated products and enzyme. The samples were then incubated with the RCA Mix (1 U/μl NxGen Phi29 DNA polymerase (Lucigen), 250 μM dNTPs, 200 μg/ml BSA (New England Biolabs), 40 μM Amino-Allyl dUTP (Invitrogen)) in 1× Phi29 DNA Polymerase Buffer for 2 h at 37°C followed by three 5-min washes with 2× SSC buffer. Finally, the samples were stained with 500 nM Cy3 or Cy5 labelled fluorescent probes in 6× SSC for 30 min, washed with 6× SSC to remove unbound fluorescent probes.

### Generation of cerebral organoids

Cerebral organoids were differentiated from human iPSCs (MH0185983) established elsewhere (18) using a previously described protocol with modifications (19). Approximately 900 000 iPSCs were seeded into a 96-well ultra-low attachment plates (Corning) with 150 μl of mTeSR1 (Stemcell Technologies) and 50 μM ROCK inhibitor (Santa Cruz Biotechnology), to obtain embryoid bodies (EBs). The media was replaced with 200 μl of neural induction media on day 4 when EBs were about 600 μm in diameter. After 3 days, 100 μl of the media in each well was replaced. On day 9, each organoid was placed on parafilm compression, and 35 μl of Matrigel (Corning, 354234) was used for embedding. About 10 embedded organoids were transferred into one six-well of an ultra-low attachment plate (Thermo Fisher Scientific) containing 5 ml of differentiation media (without vitamin A) with 1% antibiotic-antimycotic (penicillin, streptomycin and amphotericin B). On day 15, media was changed to differentiation media containing vitamin A and 1% antibiotic–antimycotic, and plates were placed on an orbital shaker at 90 rpm in the incubator at 37°C.

### Organoid sectioning

46-day old organoids were fixed overnight at 4°C on a shaker followed by 3× 30 min washes with PBS at room temperature. Subsequently, fixed organoids were embedded in optimal cutting temperature (OCT) compound followed by rapid freezing on a metal block semi-submerged in liquid nitrogen. The frozen OCT blocks were trimmed and sectioned at 20 μm thickness onto Superfrost Plus slides using the Microm HM505E cryostat.

### Library preparation protocol for organoids

Slides containing two 20 μm thick organoid sections were dehydrated with increasing amounts of ethanol from 70 to 100% and left in 100% ethanol for 1 h at room temperature. Barriers were drawn around sections using a hydrophobic pap pen, then sections were permeabilized in Triton X-100 in PBS for 15 min at room temperature. Sections were hybridized with probes in hybridization buffer (10% formamide, 6× SSC), with 100 μM probes and 200 μM RCA primer for 1 h at 37°C.

Next, the sections were rinsed once with SplintR Ligase buffer and then ligated with SplintR Ligase (New England Biolabs) for 1 h at room temperature. After ligation, sections were rinsed in Phi29 buffer three times, then incubated in Phi29 RCA mixture containing Phi29 (Lucigen), Phi29 buffer (Lucigen), 10 mM dNTPs, 2 mM aminoallyl-dUTPs, 20 mg/ml BSA and water at room temperature overnight. The next day, sections were rinsed in 10 nM MES buffer (Alfa Aesar) solution and then crosslinked for 1 h at room temp with 50 mM HEPES buffered saline, and BS(PEG)_9_. After 1 h, 20 μl 1 M Borate buffer was added to each section for 15 additional minutes. Sections were then washed for 30 min with 1 M Tris and rinsed three times with PBS. Then FISH probes were added in a solution of hybridization buffer at a final concentration of 0.5 μM FISH probes with 1 μg/ml DAPI (Invitrogen). Sections were covered from light and incubated for 30 min at room temperature. Finally, sections were rinsed three times with hybridization buffer to wash off probes and then were ready for imaging.

### Imaging organoids

Images were taken on a Nikon Ni-E upright fluorescence microscope using a LED fluorescent light source (Sola SE2). Images were obtained using a CFI Plan Apo Lambda 10× objective (NA = 0.45) and a CFI Plan Apo VC 60× water immersion objective (NA = 1.2). Images were obtained with a Hamamatsu Flash 4 v3 sCMOS camera using Nikon Elements imaging software.

### Single molecule FISH (smFISH)

smFISH probes for *GAPDH* (SMF-2026-1), *POLR2A* (SMF-2003-1) and *TFRC* (SMF-2006-1) were acquired from Stellaris. smFISH was performed according to manufacturer's instructions (‘Stellaris RNA FISH Protocol for Adherent Cells’). Cells were cultured on removable chambered coverglass (Grace Bio-Labs, 112358) prior to fixation of the smFISH samples.

### Image acquisition for low-plex BOLORAMIS and smFISH

Images were acquired on a Nikon Ti2 Eclipse inverted microscope using a Plan Apo Lambda DM 60× (1.4 NA, Ph3) oil objective and an Andor Zyla sCMOS camera. Images were acquired using the NIS-Element AR software.

### Amplicon and puncta quantification for low-plex BOLORAMIS and smFISH

For optimization of probe configurations, specificity and sensitivity test, BOLORAMIS amplicons and smFISH puncta were quantified in Fiji. Image stacks were combined by maximum intensity projection, followed by background subtraction, with a rolling ball radius of 10 for BOLORAMIS and 2 for smFISH. Images were also smoothed by default settings in Fiji. To quantify the number of spots in each cell, single cells were manually cropped out and spots were counted using the 2D/3D particle tracking function in the Mosaic plugin.

### TDR calculation for optimizing probe configuration

A set of 11 probes targeting ACTB mRNA was designed such that the 5′ hybridization arm length was systematically varied at two base intervals. The total hybridization region was kept constant at 25 nt, and the same ligation junction (dA/A) was maintained for all sets. As a control, another set of 11 probes targeting *ACTB* was designed with only one base mismatch at the 3′ ligation junction (A>T). Targeted detection was performed as follows: Confluent cultures of HeLa-Cas9 cells (Genecopoeia, SL503) were treated with 0.05% trypsin–EDTA (Life Technologies), re-suspended in 1× DMEM (Invitrogen) and seeded on six channel flowcells (μ-Slide VI0.4 ibiTreat, Ibidi). BOLORAMIS library preparation and image quantification were performed as described above. True discovery rate (TDR) is defined as the percentage of total signal observed from a perfectly matched (M) probe as compared with the sum signal of a matched and mismatched (MM) probe calculated as (M/(M + MM)) × 100. For example, if a matched probe results in a mean count of 99 spots/cell, and a mismatched probe results in a mean count of 1 spot/cell, then the TDR is 99%.

### Bulk RNA sequencing library preparation

HMC3 cells cultured in six-well plates were treated with 600 μl TRIzol (Thermo Fisher Scientific, 15596018) directly on the plate. The solution was used for total RNA extraction using Direct-zol RNA MiniPrep Kit (Zymo Research, R2050). RNA amount was quantified using Qubit RNA HS Assay Kit (Thermo Fisher Scientific, Q32852) and RNA integrity was checked by the presence of 18S and 28S bands on a 2% E-Gel EX Agarose Gel (Thermo Fisher Scientific, G402002). 1 μg RNA was used for library preparation using NEBNext Ultra II Directional RNA Library Prep Kit (New England Biolabs, E7760S) following the manufacturer's instructions on combined usage with NEBNext Poly(A) mRNA Magnetic Isolation Module (New England Biolabs, E7490). Library QC and sequencing was performed by the Biopolymers Facility at Harvard Medical School.

### Analysis of RNA sequencing data

A human genome reference index was built by STAR 2.5.2b using genome primary assembly (ftp://ftp.ebi.ac.uk/pub/databases/gencode/Gencode_human/release_27/GRCh38.primary_assembly.genome.fa.gz) and annotation file (ftp://ftp.ebi.ac.uk/pub/databases/gencode/Gencode_human/release_27/gencode.v27.primary_assembly.annotation.gtf.gz) from GENCODE. Read alignment and quantification were performed using STAR -quantMode GeneCounts. Raw RNA-seq data for day 4 iNGN were acquired from GEO (https://www.ncbi.nlm.nih.gov/geo/query/acc.cgi?acc=GSE60548). HMC3 and iNGN gene expression counts were normalized by the total aligned reads number per sample. Differential gene expression analysis was performed using DESeq2 (20).

### BOLORAMIS probe design

All mRNA-targeting probes were 60 nucleotides (nt), with a 25-nt RNA targeting region and a 35-nt barcoded linker. The barcoded linker sequence consisted of a universal 23-nt sequencing anchor, flanked by two 6-base filler sequences (Supplementary Table S1). For the 96-plex experiment, the two 6-base filler sequences were modified to be an 8-base barcode downstream of the sequencing adapter and a 4-base barcode upstream that is identical to the last 4 nt of the 8-base barcode. Because nonamer sequencing was shown to be more reliable within 4 bases (21) from either direction, the 4-base barcode on one side was copied from the 5–8 position of the 8-base barcode on the other side. This design would allow future attempts of sequencing using SOLiD or Illumina SBS chemistry, which only goes from 5′ to 3′ direction. The sequencing anchor was used for FISH and *in situ* sequencing. Phosphorylated probes were ordered from IDT in 10 nanomoles scale, at a stock concentration of 100 μM in plate format, allowing rapid design-build-test cycles.

To design specific and efficient probes for the 96-plex experiment, factors considered included off-target sequence in the transcriptome, ligation junction compatibility with SplintR (22) and probe secondary structure. Target isoforms were manually picked to be the dominant isoform according to Ensembl Genome Browser and broken up into all possible 25-mers, avoiding homotetrapolymers of guanine. Updated code available at https://github.com/pawlowac/BoloramisProbeDesign now avoids homotetrapolymers of cytosine as well. A reference database was created from the Human Gencode (23) GRCh38 release 29 by removing any genes with baseMean < 10 using DESeq2 (20) found during the RNA-seq experiment described above, leaving 15 596 out of 56 653 genes to search for potential off target hybridization of BOLORAMIS probes. Off-target hybridization was evaluated with Bowtie2 (24) v1.2.3 with the following flags; -D 20 -R 3 -N 1 -L 9 -i L,0,0.8 –gbar 13 -k 50000 –score-min C,-42,0. Bowtie2 results were parsed for probes with at least six mismatches to a non-target mRNA. Secondary structure can have an impact on target hybridization and probe ligation, so potential secondary structure of the final probe sequence (hybridization arms + adapter + barcode) was predicted using RNAfold (25) v2.4.11 and the dna_matthews2004.par (26) model for DNA. RNAfold was used with the following flags; -p -d2 –noLP –noPS —noconv –MEA. Probes were ranked by the free energy of secondary structure, and the top 10 probes with the least secondary structure were chosen if >10 probes were found. Probes were ordered with a 5′ phosphate required for SplintR ligation.

### Barcode design with Hamming distances and error correction

8-base barcodes with a Hamming distance of 3 were designed using the create.dnabarcodes (8, dist = 3) function in DNABarcodes R package (27). For error correction, all non-perfect matching barcodes were calculated for their Hamming distance to all 173 reference barcodes. Any barcode with a minimum distance >2 to reference barcodes were discarded. Any barcode with a non-unique minimum distance to reference barcodes were also discarded. All non-perfect matching barcodes with a unique minimum distance of one or two to one of the reference barcodes were assigned the identity of that reference barcode.

### Nonamer *in situ* sequencing-by-ligation

Nonamer sequencing was performed following published protocol (21) with modifications. To minimize amplicon movement during eight rounds of *in situ* sequencing (Supplementary Figure S8), amplicons were fixed using BS(PEG)_9_ (Thermo Fisher Scientific, 21582) by crosslinking incorporated aminoallyl-dUTP (Thermo Fisher Scientific, R1101) with surrounding proteins. After RCA, the sample was gently rinsed once with 10 mM MES, pH 6.5. Amplicon fixation buffer (for 200 μl, use 172 μl water, 4 μl 500 mM MES, pH 6.5, 4 μl BS(PEG)_9_, spike in 20 μl 1 M Borate halfway at 15 min) was added to the sample for 30 min at room temperature. Fixation reaction was quenched by 1 M Tris–HCl, pH 8.0 and sample was rinsed three times with 1× PBS.

For nonamer sequencing, all steps were performed on a microscope stage where the sample was fixed in place. All steps were performed at room temperature unless specified otherwise. Sample was incubated with sequencing anchor mix (1:100 dilution, anchor stock 100 μM, in hybridization buffer (6× SSC + 10% formamide)) for 15 min, followed by three 1-min washes with 6× SSC and one rinse with 1× T4 Ligase buffer. Nonamer mix (for 200 μl, use 168 μl water, 10 μl T4 DNA Ligase, 2 μl 100 μM nonamer stock, 20 μl 10× T4 Ligase buffer) was added to the sample for 45–60 min, followed by three 1-min washes with 6× SSC. Sample was imaged for this sequencing position. Nonamers were stripped away by incubating with stripping buffer (for 1000 μl, use 800 μl formamide, 10 μl 0.01% Triton-X-100, 190 μl water) preheated to 80°C three times for 3 min each. Three rinses with 6× SSC was performed to remove residual formamide and the sample was ready for the next sequencing round.

### Image acquisition for *in situ* sequencing

*In situ* sequencing was performed on a Nikon Ti-E Inverted Microscope with PFS3 Yokogawa CSU-X1 spinning disc confocal (Nikon). Sample was fixed on the microscope stage during the eight rounds of nonamer sequencing. Sample was imaged with a 40× NA 1.1 CFI Lambda S Apo LWD 4 Water Immersion Objective Lens (Nikon, MRD77410). Each field of view (FOV) was 1600 × 2048 px with a 20% overlap between tiles in a 5 × 5 tile scan. 63 z-stacks with a 500-nm step size were taken for each FOV. LUNV NIDAQ lasers (488, 561, 594, 640 nm) with 8900 Sedat Quad dichroic mirrors (425–477, 503–542, 571–628 and 661–728 nm) were used to distinguish the four fluorophores (FAM, Cy3, Texas-Red, Cy5) in the nonamer mix.

### *In situ* sequencing data pre-processing

*In situ* sequencing data analysis were performed as described in Alon *et al.* (28). Briefly, the pipeline included four major steps: feature-based 3D registration, background subtraction, amplicon segmentation, and basecalling. Feature-based 3D registration was conducted in three steps: keypoint detection, feature construction at a keypoint, and feature matching. The matched features were then used to create corresponding points between different images, which were then used to calculate a warp of the processed image into the coordinate space of the reference image. Registered imaged were then manually inspected in FIJI and the non-uniform background signal were subtracted by applying a morphological opening operation of 5-pixel size, which was subtracted from the original image. Any negative values were converted to zero. To identify amplicons produced by RCA, a watershed transform method was used. To increase the signal, grayscale 3D images after registration and background subtraction from all *in situ* sequencing rounds were summed to create a composite image. This composite image was interpolated via a shape-preserving piecewise cubic interpolation in Z and punctate signal was amplified using a Difference of Gaussians filter. The filtered image was binarized using the Otsu method and segmented using the watershed transform. The segmented image was uninterpolated in Z back into the original image dimension. For basecalling, the pixels for the four-color channels in each sequencing round were quantile normalized to account for different fluorophore characteristics. For each amplicon, the normalize intensities for each channel were sorted and the averages of top 30 pixels were compared. The highest channel was selected as the color identity of this amplicon in this *in situ* sequencing round. After all eight sequencing rounds were basecalled, an eight-base barcode was concatenated for each amplicon and mapped to the reference barcode list.

### Cell segmentation

Cell segmentation was performed on z-projected, stitched DAPI (nucleus) + FISH (all amplicon) images according to previous description (29). Briefly, the raw confocal image was first processed with a gray morphology top-hat filter to reduce background noise. To assign individual amplicons to individual cell nuclei, DAPI channel was segmented by first extracting the tensor structure energy, then by binary segmentation and water-shedding. The perinuclear zones were then obtained by sequential dilatations on the binary segmented cell nuclei. Cell nuclei were then assigned to perinuclear zones by a point-in-polygon algorithm. The amplicon number, position within perinuclear zone, and position of a given cell nuclei were used as input for an affinity propagation clustering algorithm (30), with a negative-squared-distances similarity measure. The initial number of clusters was set to the number of segmented cell nuclei using the apclusterK function in the apcluster R package (31).

### Single-cell spatial analysis

Single-cell spatial analysis was performed with Giotto R package (32). Gene and cell filters used in this study were: expression_threshold = 1, gene_det_in_min_cells = 5, min_det_genes_per_cell = 15. For clustering, shared nearest neighbour (sNN) network was created with 5 dimensions and 15 neighbors. A Leiden community detection algorithm was used on the sNN-network with 0.1 or 0.2 resolution and 100 iterations. Cluster markers were identified by using MAST method on the scaled expression matrix with a *P*-value threshold of 0.01 and log FC threshold of 0.5. For Delaunay spatial network creation, distance cutoff for the nearest neighbors was 400 and minimum nearest neighbor was 2.

## RESULTS

### Optimization of BOLORAMIS probe design

The workflow of BOLORAMIS consists of the following steps (Figure 1A): (i) gene selection and probe design; (ii) library preparation; (iii) FISH or *in situ* sequencing; (iv) image acquisition and quantification. First, genes are selected and probes to these genes are designed using our software. Next, library preparation is performed on a cell line of interest. During library preparation, cells are fixed and permeabilized, followed by hybridization of probes and RCA primers. Hybridized probes to the target are then circularized with SplintR Ligase to create RCA templates. The circular templates are amplified using the highly processive Phi29 DNA polymerase to produce amplicons. Amplicons are then visualized by FISH or *in situ* sequencing depending on the study design. Image acquisition of the amplicons observed as puncta in each cell is done and the puncta number is quantified.

To demonstrate that BOLORAMIS library preparation works on diverse sample types, we targeted abundant housekeeping genes like *ACTB* in cell lines (HeLa, MCF7, HEK293, NIH-3T3) (Figure 1B) and *GAPDH* in sections of human cerebral organoids (18,19) (Figure 1C). After library preparation and FISH (Materials and Methods), dense amplicons generated from RCA of the targeted probes bound to these abundant genes were observed in all sample types, demonstrating the broad applicability of BOLORAMIS. Previous studies on SplintR-mediated ligation showed that ligation efficiency and specificity could be affected by the length of 5′ and 3′ hybridization arms (15,22). The hybridization length used in this study was 25 nt; together with 23 nt of RCA primer and 12 nt of barcode, the total length of our probe is 60 nt. This design is long enough for sequence-specific detection (data below) and short enough for cost-friendly synthesis where generally there is a large increase in cost from ≤60 nt to >60 nt. To our knowledge, our probe length is the shortest compared with previously published work on SplintR-based *in situ* detection, which are around 90 nt (33–35).

To determine the best position for placing the ligation junction within the 25 nt region, we designed probes targeting *ACTB* with 5′ hybridization arms ranging from 2 nt to 22 nt, together with their negative control probes with a single mismatch at the ligation junction targeting the exact same region of the *ACTB* gene (Supplementary Table S1). We quantified the number of amplicons per cell to assess the relative efficiency of these positions and calculated true discovery rate (TDR, Materials and Methods and Supplementary Table S4) to assess their accuracy. The probe that produced maximal amplicon and also had a high TDR value was the one which had 18 nt on the 5′ arms and 7 nt on the 3′ arms (Figure 1D–F; Supplementary Figure S1). This probe configuration was used for downstream studies. It is worth noting that even when targeting a gene as abundant as *ACTB*, a single mismatch at the ligation is enough to abolish over 95% of the signal in the 18 + 7 nt group. This indicates that if the ligation junction is placed exactly upon a point mutation within a transcript, BOLORAMIS probes have the potential to detect the single point mutation.

### Singleplex detection of coding and non-coding RNAs using individual probes

To determine if BOLORAMIS can work on a wide range of RNA targets in addition to abundant genes such as *ACTB* and *GAPDH*, we evaluated BOLORAMIS’ performance in detecting coding and non-coding RNA targets, individually, in a biologically meaningful context. We designed 77 probes targeting 77 miRNAs and 192 probes targeting 77 mRNA of transcription factors (TFs), expressed at varying abundance levels in human iPSCs (Supplementary Methods, Supplementary Table S2) (16). Multiple probes targeting different regions of the same TF transcripts were designed to evaluate the variability in detection. Because the mature miRNAs we chose to target have a length between 18–23 nt, which is shorter than 25 nt, the following parameters were applied during probe design: (i) that the entire length of the miRNA (18–23 nt) is used for targeting; (ii) that the 3′ arm is at least 7 nt and (iii) that poor ligation junctions are avoided, as listed in Supplementary Figure S7. Each probe was tested individually *in situ* in PGP1 human iPSCs in 384-well plates. Single-cell spot counts were quantified from a total of 217 206 human iPSCs using automated imaging and an image-processing pipeline (Supplementary Figure S2, Supplementary Methods).

On the whole, BOLORAMIS expression values in human iPSCs were indicative of a stem cell expression signature (Supplementary Figure S3). Of note, some of the highest ranked mRNAs corresponded to pluripotency markers (*ZFP42*, *GATA2*, *SMAD1*, *ID1*, *KAT7*, *OCT4*, *SOX2*, *NANOG* and *ZFX*) (Supplementary Figure S3A). Conversely, mRNAs with the lowest BOLORAMIS single-cell expression values were associated with promoting cellular differentiation. For example, the lowest ranked TFs were *NR61A*, *NEUROG1*, *LIN28B*, *TAL1*, *NR4A2*, *CREB1*, *OLIG2* and *NEUROG2* (Supplementary Figure S3A). Example microscopy images used for quantification are shown in Supplementary Figure S4. Single cell BOLORAMIS expression counts varied between 1–1000 spots/cell with a mean of 53.8 ± 74.9 SD. The highest detected mean BOLORAMIS expression value was 236.95 amplicon/cell (*ZFP42*, a pluripotency gene, Supplementary Figure S3B), which is comparable with the reported upper-limit of high-throughput branched DNA (bDNA) smFISH detection at similar magnification (36,37).

We next compared BOLORAMIS mRNA measurements with published RNA-seq values. Overall, BOLORAMIS probes exhibited a low-positive correlation with bulk RNA-seq values (Pearson's *r*: 0.142). We suspect that the low correlation might likely be resulting from the variable efficiency between single probes to detect their targets. To test this hypothesis, we calculated the coefficient of variation (COV) of amplicon number generated by probes targeting different regions of the same transcript. The mean COV between at least two independent probes targeting the same RNA was 52.63 ± 29.46% (SD, *n* = 53, Supplementary Figure S3D). One factor previously reported to affect SplintR Ligase activity was the bases near the ligation junction (22). To investigate if differences in ligation junction could contribute to the variability of detection, we looked at correlation with bulk RNA-seq values while grouping probes by their ligation junction sequences (Supplementary Figure S3H, Supplementary Table S15). We observed a wide range of Pearson correlation values ranging from -0.3 to 0.9. The three lowest correlation junctions (5′/3′) were TG (*n* = 16), CG (*n* = 9) and GA (*n* = 19), which is consistent with previous reports about poor ligation activity on junctions containing G. The three highest correlation junctions were TC (*n* = 10), CC (*n* = 4) and AC (*n* = 13). While it may seem reasonable to prioritize these junctions in future experiments, we would like to point out that the limited size of the sample and other factors could also affect probe efficiency, including sequences surrounding the actual junction bases (22), melting temperature of the 25 nt targeting region (Supplementary Table S14), local secondary structures of the transcripts, and bound proteins that could block hybridization. With these in mind, we suggest that even though BOLORAMIS can work with a single probe, to accurately determine the relative expression level of mRNAs, it would be beneficial to pool multiple probes for the detection of the same transcript. We used this format of using multiple probes per transcript detection for downstream experiments.

Due to RNA size constrains and variations, miRNA can only be targeted with one probe and we prioritized ligation junction compatibility with SplintR (22) over the 18/7 hybridization arm design. We selected 77 miRNAs with published bulk NanoString nCounter expression measurements in PGP1 human iPSC (16) (Supplementary Figure S3C). BOLORAMIS count of 77 miRNAs exhibited a poor correlation with published bulk measurements (Pearson's *r*: 0.067). However, for a given probe, we observed mostly reproducible mean spot counts per cell for independent BOLORAMIS assays (Pearson's *r*: 0.62, Supplementary Figure S3F, G). This suggested that although the experiment is reproducible, the relative miRNA expression level determined by BOLORAMIS is not consistent with bulk measurements (Discussion).

### Determining specificity and sensitivity of BOLORAMIS

**Figure 2.**
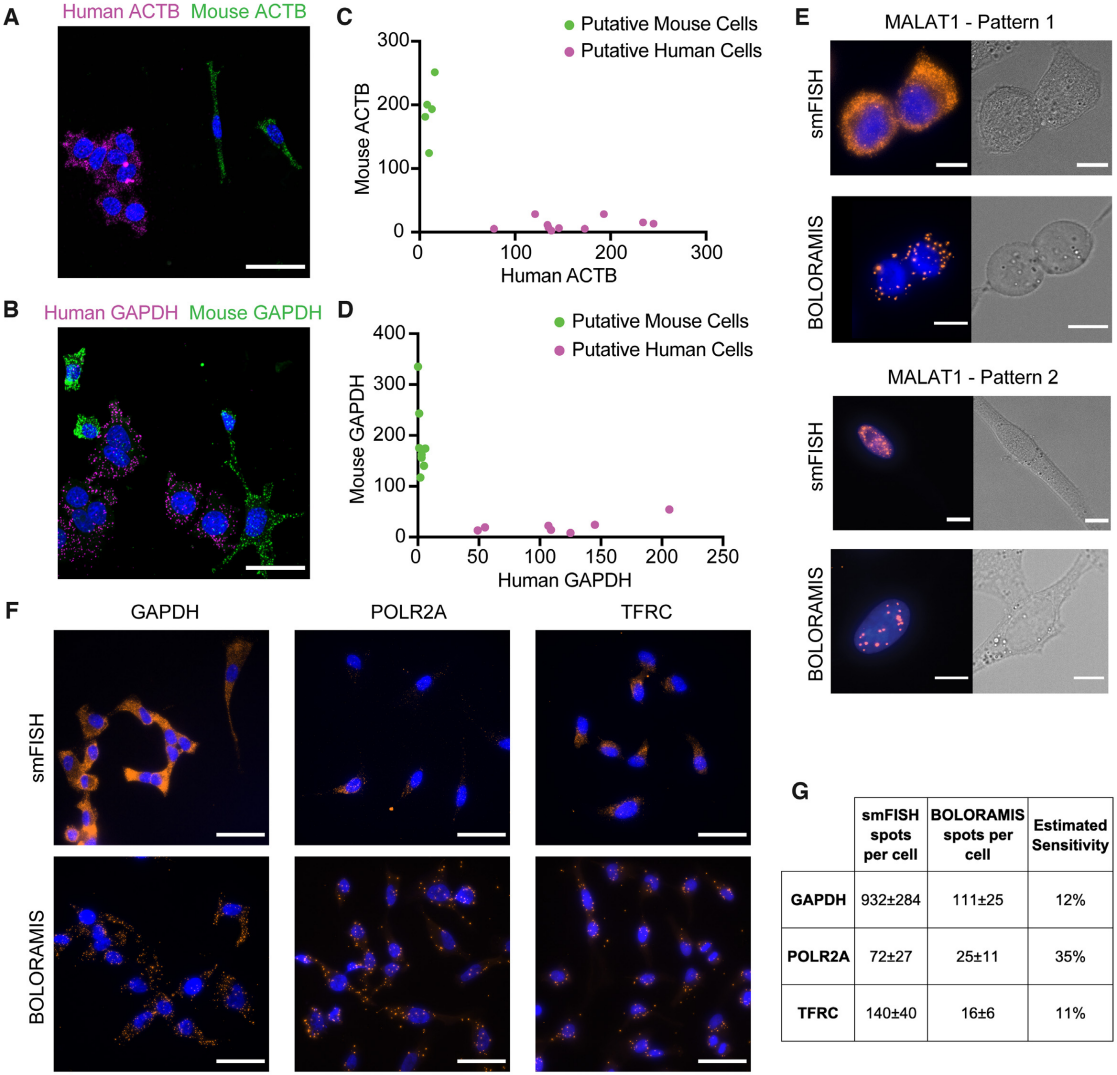
Specificity and sensitivity of BOLORAMIS. (**A**–**D**) Specific detection of *ACTB* (*n* = 15) and *GAPDH* (*n* = 15) in the co-culture of MCF7 (human, scale bar: 50 μm) and NIH-3T3 (mouse, scale bar: 50 μm) cells. (**E**) Subcellular localization of lncRNA *MALAT1* detected by smFISH and BOLORAMIS (scale bar: 10 μm). Left, cytoplasmic; right, nuclear. (**F**) Images for side-by-side comparison of smFISH and BOLORAMIS for detecting the same genes in HeLa cells (scale bar: 50 μm). (**G**) Quantitative comparison with smFISH in HeLa cells for detection of *GAPDH* (*n* = number of cells, smFISH *n* = 6, BOLORAMIS *n* = 9), *POLR2A* (smFISH *n* = 7, BOLORAMIS *n* = 17) and *TFRC* (smFISH *n* = 11, BOLORAMIS *n* = 22).

To show that transcripts with high sequence similarity can be distinguished by BOLORAMIS before performing multiplexed *in situ* sequencing experiments, we designed probes that specifically targeted human or mouse genes (*ACTB* and *GAPDH*). Each transcript was targeted with a pool of three probes at different and specific regions on ACTB and GAPDH mRNA (Supplementary Tables S1 and S5). For each gene, three probes each targeting human and mouse transcripts were pooled together and used for BOLORAMIS library preparation in a co-culture of human MCF7 and mouse NIH-3T3 cell lines. We observed clear separation of human and mouse targets based on the FISH-stained images (Figure 2A–D). In putative mouse cells, 94.6 ± 1.8% (*n* = 5) *ACTB* amplicons, and 98.4 ± 1.3% (*n* = 8) *GAPDH* amplicons were detected for mouse *Actb* and *Gapdh* respectively; while in putative human cells, 93.1 ± 5.2% (*n* = 10) *ACTB* amplicons and 83.4 ± 6.6% (*n* = 7) *GAPDH* amplicons were observed for human *ACTB* and *GAPDH* respectively (Supplementary Table S6). These results indicated probe specificity between transcripts that are highly similar in their gene sequences. BOLORAMIS was also able to specifically detect subcellular localization of transcripts. We observed long non-coding RNA (lncRNA) *MALAT1* exhibiting two distinct subcellular localization patterns in HeLa cell cultures, where in each cell *MALAT1* was found in either the nucleus or in the cytoplasm (Figure 2E). This observation was confirmed by smFISH (Figure 2E) and has also been demonstrated by previous studies (38,39).

To determine the sensitivity of BOLORAMIS, we compared our method with smFISH, which is considered as the ‘gold standard’ for *in situ* molecule counting. We targeted three genes with validated commercially available smFISH probes (Stellaris): *GAPDH*, *POLR2A* and *TFRC*. These three genes were chosen such that they had variable expression levels and the probes were readily available for shipping. Each transcript was targeted with a probe pool at ten different and specific regions on the mRNA and BOLORAMIS was performed in HeLa cells (Supplementary Table S1, Figure 2F). The number of spots per cell was counted for the two methods, and sensitivity was calculated by dividing the average number of spots per cell from BOLORAMIS by that from smFISH. The detection sensitivity of BOLORAMIS compared to smFISH for the three genes was 11% (*GAPDH*), 35% (*POLR2A*) and 12% (*TFRC*), respectively (Figure 2G, Supplementary Table S7). The differences observed between sensitivity of smFISH and BOLORAMIS could be a result of additional enzymatic steps used in BOLORAMIS protocol, including SplintR ligase-mediated probe circularization and Phi29 polymerase-mediated RCA. However, the sensitivity of our method is still comparable to the widely used high throughput single-cell RNA sequencing technologies, which is around 10–30% (40,41). Additionally, BOLORAMIS has potential for highly multiplexed detection, whereas conventional smFISH is limited to 3–5 targets.

Before performing a multiplexed experiment using BOLORAMIS, we wanted to investigate how the accuracy of detection changed with increased number of probes per gene. To test this, we picked eight genes with different expression levels based on bulk RNA sequencing data from MCF7 cells and PGP1 human fibroblasts: *CSDE1*, *SPTSSB*, *DDX5*, *SLC39A6*, *EEF1A2*, *CALR*, *FLNA* and *TFRC*. Using our automated probe design software (Materials and Methods, Supplementary Figure S7), we designed and synthesized 2, 5, 10 and 24 probes for each gene. The four probe pools for the eight genes were tested in duplicates in each cell type (Supplementary Figure S5, S6A, B). After image quantification, we observed a clear trend of increase in puncta per cell for all eight genes as the number of probes increased (Supplementary Figure S5A, C, S6A, B). Of note, in all samples the total probe concentrations were kept the same at 1 μM, indicating a more diverse probe pool is likely to yield a better detection for the gene. To test if the relative expression levels between genes are better captured by BOLORAMIS when more probes are used per gene, we correlated the mean puncta number per cell for the eight genes with their corresponding transcripts per million (TPM) values from bulk RNA sequencing. In both MCF7 cells and PGP1-fibroblasts, we observed an increased Pearson correlation coefficient with bulk RNA-seq when probes per gene increased from 2 to 5. However, we did not observe a further increase in correlation when 10 or 24 probes were used (Supplementary Figure S5B, D). The correlation coefficient hovered around 0.5 even when 24 probes were used per gene, indicating the limitation of BOLORAMIS for accurate quantification of gene expression. Even though BOLORAMIS detection is feasible with a single probe, as demonstrated earlier on *ACTB*, we recommend using 5–10 probes per gene as a test starting point when designing probes for new experiments.

To assess the reproducibility to BOLORAMIS detection, we looked at puncta number per cell from the duplicated samples from the above experiments for eight genes (Supplementary Figure S6A, B), as well as a triplicated measurement for two genes in two cell lines (Supplementary Figure S6C, D). In both experiments, we observed overall high consistency between replicates, indicating good reproducibility of BOLORAMIS detection.

### Demonstrating multiplexing of BOLORAMIS and determining spatial gene expression patterns

Having optimized the probe configuration, benchmarked the specificity and sensitivity, we set out to study single-cell and spatial heterogeneity of cells in a neuron/microglia co-culture. Microglia play important roles in brain development and disease (42). Microglia have been implicated to play major roles in neurodegenerative diseases, such as Alzheimer's disease, through their tight link between inflammation and neuron survival (43,44). We wanted to determine if the interplay between neuron and microglia could be partially replicated in an *in vitro* co-culture experiment.

**Figure 3.**
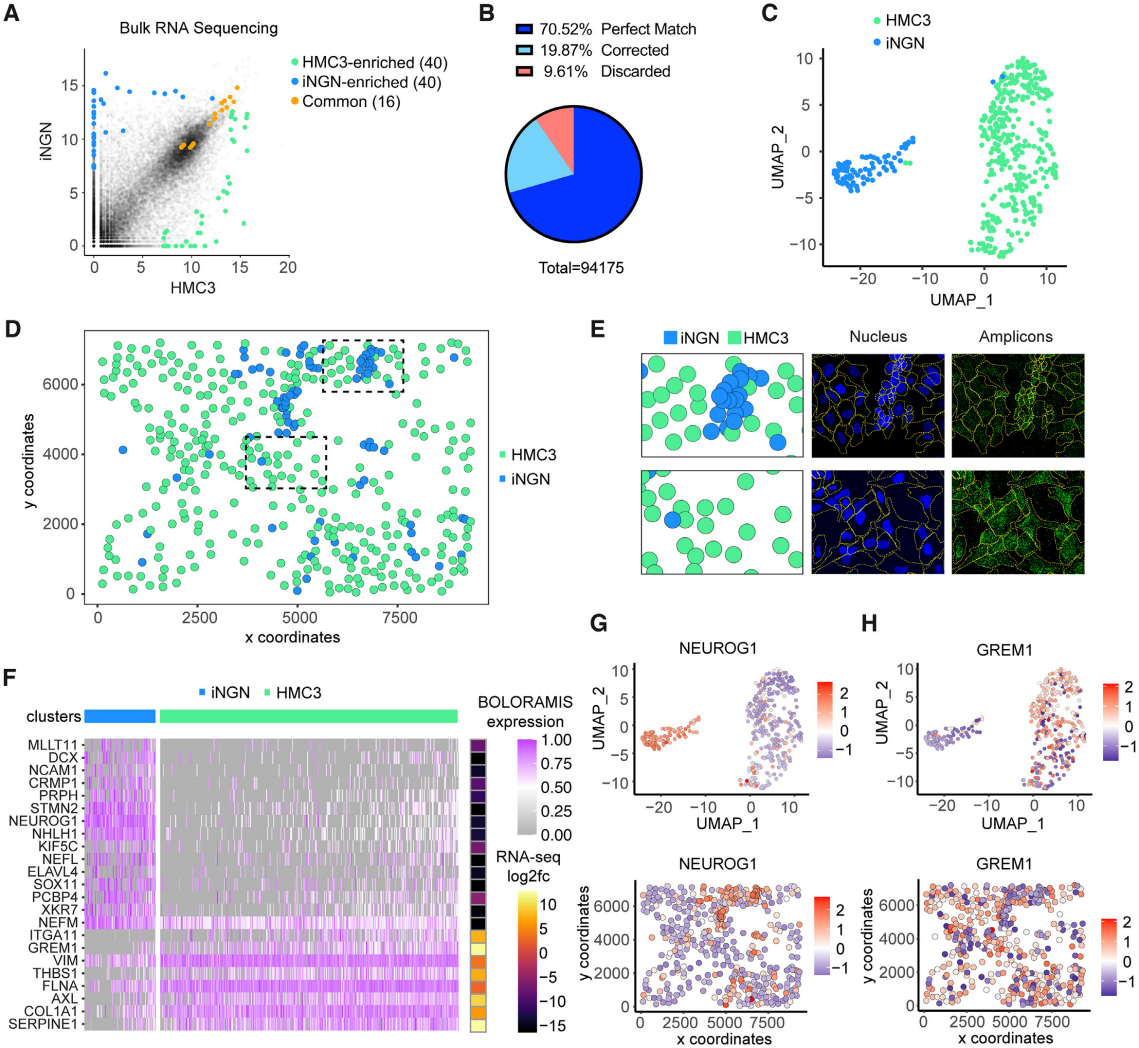
Multiplexed detection of 96 genes in a co-culture of HMC3 and iNGN. (**A**) Selection of 96 genes from differential gene expression analysis (blue, iNGN enriched; green: HMC3 enriched; Orange: shared). (**B**) Statistics for error correction of barcodes. (**C**) Clustering analysis of segmented single cells (Leiden resolution = 0.1). (**D**) Spatial visualization of segmented cells with their predicted cell type. Dashed box zoomed in panel E. (**E**) Side-by-side comparison of classified single cells and segmented nucleus + amplicon images. (**F**) Cluster markers identified by MAST, side-by-side with log_2_fc values from bulk RNA-seq data, indicating consistency between BOLORAMIS and RNA-seq for cell marker identification. (**G**, **H**) UMAP and spatial visualization of *NEUROG1* (iNGN-enriched), and *GREM1* (HMC3-enriched).

To demonstrate the ability of BOLORAMIS to study single-cell and spatial patterns of the transcriptome, we targeted 96 mRNAs simultaneously in a co-culture of iNGN neurons (16) and a human microglial cell line (HMC3) (17). Based on differential gene expression analysis of bulk RNA sequencing data from the monoculture of iNGN and HMC3, the 96 genes were selected to include 40 iNGN-enriched genes, 16 common genes and 40 HMC3-enriched genes (Figure 3A, Supplementary Table S3,8). The genes span a range of >600-fold difference in their expression levels, with DESeq2 (20) baseMean value ranging from the lowest 64 (*TLR4*) to the highest 39652 (*NEFM*), with *ACTB*’s value being 76 117 as a reference (Materials and Methods). Similar to the smFISH comparison experiment, most of the transcripts were targeted with 9–10 probes with the common 8-nt barcode. In addition to the 96 mRNAs, we also targeted 77 miRNAs with known expression data in iNGN (16) to assess the ability of our current BOLORAMIS protocol on miRNA detection. Thus, a total of 957 mRNA probes + 77 miRNA probes (Supplementary Table S3) with 96 unique mRNA barcodes (one barcode per gene) + 77 miRNA barcodes were pooled in this multiplexing experiment. The specificity, ligation junction and secondary structure of the probes were checked using our custom probe design software (Materials and Methods, Supplementary Figure S7). We used this probe set to perform BOLORAMIS on the co-culture of iNGN and HMC3 cells. After library preparation, the sample was sequenced *in situ* for 8 cycles using nonamer sequencing-by-ligation (21) (Supplementary Figure S8).

After image registration, spot identification, base-calling and barcode identification (Materials and Methods), 94 175 amplicons were identified in an area of 800 μm by 1200 μm. 70.5% (66 410) of the amplicons had a perfect match to the reference barcode list (Supplementary Table S9 and S10). The barcodes were designed to be robust to sequencing errors by enforcing a Hamming distance of 3 (27). We therefore identified single and double nucleotide errors and recovered 18 715 additional amplicon barcode identities (Materials and Methods, Figure 3B). Among identified amplicons, we observed non-zero counts for all 96 mRNA targets (Supplementary Figure S9), with the highest count for *NEFM* (12701 amplicons) and lowest count for *KLF2* (2 amplicons). Of note, among the 66 410 perfect matching barcodes, only 10 (<0.02%) were miRNA barcodes, with a count of 1 for 10 different miRNAs. However, among the 18,715 barcodes recovered from error correction, 1244 (6.6%) were miRNA barcodes. The reason for overrepresented erroneous barcodes for potential miRNA reads (Supplementary Figure S9) remains to be determined. Due to the anomalous results of miRNA detection, including low counts and high barcode error rate, reads from the 77 miRNAs were excluded from downstream analysis. This data also suggested that the current iteration of BOLORAMIS protocol needs to be further optimized for miRNA detection (Discussion).

Single cells were segmented on the DAPI (nucleus) + FISH (all amplicons) image using an affinity propagation clustering algorithm (29,31). Accurate segmentation of single cells was confirmed by visual assessment (Figure 3E, Supplementary Figure S11). A cell-by-gene raw count matrix (Supplementary Table S11) and a list of cell centroids (XY coordinates, Supplementary Table S12) were used as input for single-cell spatial analysis using Giotto (32). After applying gene and cell filters (Materials and Methods), 92 mRNAs and 423 cells were used for further analysis. On average, 263 reads were acquired from each single cell, detecting 36 different mRNA targets (Supplementary Figure S10A, B). Single-cell clustering analysis identified two cell clusters (Figure 3C), which were annotated as HMC3 (340 cells) and iNGN (83 cells) according to the cluster markers identified (Figure 3F and Supplementary Figure S12D). Spatial distribution of annotated single cells is shown in Figure 3D. Markers identified for putative HMC3 cells (including *COL1A1*, *SERPINE1*, *AXL*, *FLNA*, *MYL9*, *GREM1*) were highly consistent with bulk RNA-seq data as indicated by a side-by-side comparison with log_2_fc values from DESeq2 (Figure 3F), the same was true for putative iNGN cells (including *NCAM1*, *NEUROG1*, *STMN2*, *NHLH1*, *NEFL*) (Supplementary Table S8). Cluster and spatial distribution of genes including *NEUROG1* (iNGN-enriched, Figure 3G), *GREM1* (HMC3-enriched, Figure 3H) and additional ones (Supplementary Figure S13, Supplementary Table S8) agreed with expectations based on bulk RNA-seq data. The relative expression levels of all 96 mRNA targets observed by BOLORAMIS were compared with RNA-seq expression data, showing a Pearson correlation coefficient of 0.55 before barcode error correction and 0.62 after error correction (Supplementary Figure S10). This indicates that even though BOLORAMIS is good at capturing spatial patterns of genes, the accuracy for measuring relative gene expression levels can still be improved.

To reveal spatial relationships between genes, we performed a gene-gene proximity analysis (Supplementary Methods, Supplementary Figure S14A–C). By performing an unbiased hierarchical clustering with the gene–gene proximity value matrix, two groups of genes immediately stood out to our attention: one containing *NHLH1*, *NEUROG1*, *STMN2* and *SOX11*, and the other containing *COL1A1*, *SERPINE1*, *FLNA* and *VIM* (Supplementary Figure S14D). Based on previous bulk RNA-seq and single-cell clustering results in Figure 3, 14 out of 14 mRNAs from the first group were iNGN-enriched genes, and 23 out of 35 mRNAs from the second group were HMC3-enriched genes, indicating that genes enriched in the same cell type occurred more frequently together as compared to genes that were enriched in a different cell type.

### Single-cell and spatial heterogeneity analysis of neuron/microglia co-culture

**Figure 4.**
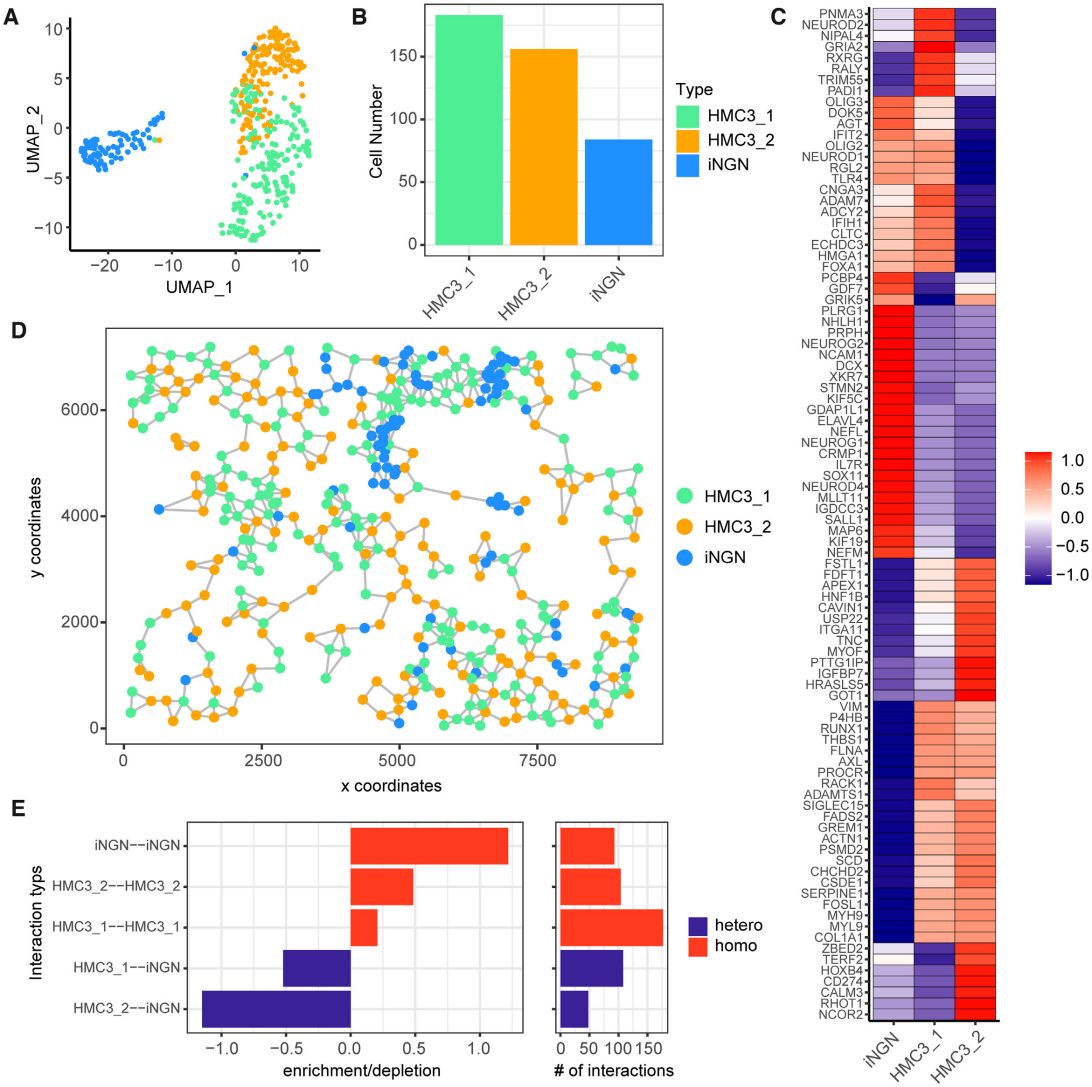
Single-cell and spatial heterogeneity in neuron/microglia co-culture revealed by BOLORAMIS. (**A**) Higher resolution of Leiden community detection (resolution = 0.2) revealed sub-clusters with HMC3 cells. (**B**) Cell number of each cluster. (**C**) *Z*-score heatmap of post-QC mRNAs expression in three clusters. (**D**) Spatial network based on cell centroid distances using Delaunay method. (**E**) Observed over expected frequency of cell-cell proximity interactions with 2000 simulations. Each simulation reshuffles the cell type labels of each node in the spatial network.

As shown in the analysis above, with a clustering resolution of 0.1, we were able to identify the two cell types (iNGN and HMC3) seeded in the co-culture experiment. To reveal heterogeneity within one cell type or to identify cell sub-types, a higher clustering resolution could be used. In order to determine if BOLORAMIS could capture heterogeneity within one cell type, we increased the resolution of cell clustering (from 0.1 to 0.2, Materials and Methods) and identified two sub-clusters within the HMC3 cluster, suggesting heterogeneity within this population (Figure 4A, B). We called the two sub-populations of the microglia ‘HMC3_1’ and ‘HMC3_2’. By comparing mRNA expressions between clusters, we identified genes that were differentially expressed between HMC3_1 and HMC3_2 (Figure 4C). Compared with HMC3_2, HMC3_1 is enriched for genes including *PNMA3*, *RXRG* and *TRIM55*, while depleted of genes including *ZBED2*, *TERF2*, and *HOXB4*. We were curious to see if spatial relationships between cells within the co-culture could be a relevant factor for this heterogeneity. To quantify the spatial relationships between cells, we built a spatial network based on cell centroid distances using the Delaunay method (32) (Figure 4D). Within this network, each node is a cell, and each edge is a cell–cell interaction. By counting the numbers of observed interactions and comparing against the simulated numbers from computationally-reshuffled network, we observed that homo-interactions (iNGN–iNGN, HMC3_2–HMC3_2, HMC3_1–HMC3_1) occurred more frequently than hetero-interactions (HMC3_1–iNGN, HMC3_2–iNGN) (Figure 4E). We also found that HMC3_1 interacted with iNGN relatively more frequently than with HMC3_2 (Figure 4E). This is especially interesting because we also observed 16 genes that had similar enrichment profile between iNGN and HMC3_1, but depleted in HMC3_2, including *OLIG3*, *NEUROD1* and *TLR4*. We ruled out the possibility of erroneous segmentation due to close proximity because many other highly expressed iNGN-enriched genes, including *NHLH1*, *NEUROG2*, *NCAM1*, were not observed in HMC3_1. The shared gene expressions between iNGN and HMC3_1 could be related to their more frequent spatial interactions. This type of analysis would be especially useful in a complex tissue setting, where cellular organization could be altered as a result of development, disease progression or drug treatment. Based on the analyses above, we concluded that BOLORAMIS was able to reveal single-cell and spatial patterns of the transcriptome.

## DISCUSSION

Spatial transcriptomics is a rapidly evolving field and the interests of obtaining 3D information of transcripts in cells, tissues, and even whole organisms have sparked the development of a number of promising technologies, including but not limited to *in situ* padlock (6), FISSEQ (5,45), ExSeq (28), MERFISH (10), seqFISH+ (12,13), slide-seq (46), HDST (47), INSTA-seq (48) and STARmap (14). In this repertoire, we have added another technology, BOLORAMIS, to the arsenal of spatial transcriptomics (Supplementary Table S16). Compared with untargeted methods, including FISSEQ, untargeted ExSeq, slide-seq, HDST and INSTA-seq, BOLORAMIS offers higher sensitivity by targeting specific genes of interest in a highly multiplexed manner. Compared with *in situ* padlock probe methods, BOLORAMIS removes the need for RT by using a RNA-splinted DNA ligase, thus reducing potential detection bias and experimental cost resulting from RT. Compared with other RT-free methods, including MERFISH, seqFISH+ and STARmap, BOLORAMIS demonstrates the shortest foot-print needed for detecting transcripts *in situ*, which is 25 nt. Although MERFISH and seqFISH+ have demonstrated superior sensitivity, amplicon-based methods like BOLORAMIS, STARmap, in situ padlock and ExSeq are more readily applicable in thick tissue samples because of stronger signals resulting from RCA. In the current study, we have demonstrated the feasibility of using the BOLORAMIS protocol on multiple cell lines and human cerebral organoids. This provides a new way of probing 3D transcriptomic architecture of organoids and opens up new opportunities for studying brain disorders, such Alzheimer's disease (49), schizophrenia and bipolar disorders (unpublished data). We have also optimized the configuration of BOLORAMIS probes to allow for a high TDR while not sacrificing detection efficiency. We show that BOLORAMIS is very specific to genes with highly similar sequences, which opens up opportunities for detecting gene isoforms and mutations. We found the sensitivity of BOLORAMIS to range between 11 and 35% when comparing spots per cell of the same gene with smFISH, which is comparable to the sensitivity of high-throughput single-cell RNA sequencing (40,41). Finally, we successfully demonstrated multiplexed *in situ* RNA detection using BOLORAMIS by targeting 96 mRNAs and revealed the spatial clustering patterns of cells in a neuron/microglia co-culture system. Hence, we believe our technology will be useful for studying spatial gene expression in more complex settings, including primary tissues and organoids.

Compared with previous reports on SplintR-based *in situ* detection methods (33–35), we have made significant improvements on probe design and sequencing chemistry to really allow for highly multiplexed *in situ* investigations. First, our probe length is only 60 nt while previously reported probes are around 90–100 nt (33–35), thereby significantly reducing the cost for probe synthesis (Supplementary Figure S15A), which makes our method more amenable to highly multiplexed experiments. We also performed a cost analysis for different strategies of probe library synthesis, including regular tube order, plate order (this study), oligo pools and array-based DNA synthesis. Based on the analysis, array-based DNA synthesis can be used to bring down the cost for genome-wide probe synthesis (Supplementary Figure S15B, Supplementary Table S13). We reported an approach to generate padlock probe library from array-synthesized DNA pools in a previous study (50). The probe cost can be further reduced by performing phosphorylation in-house using T4 polynucleotide kinase. Second, we have provided a ready-to-use automated probe design software that checks for targeting arms specificity, ligation junction compatibility with SplintR (22) and probe secondary structure. With a list of targeted transcript sequences and a reference genome, a library of probes can be designed easily and ready for synthesis. Lastly, adopting nonamer sequencing-by-ligation allowed us to encode a large diversity of targeted transcripts into barcode sequences as short as 8 nt. The upper limit of this encoding scheme is 4^8^ = 65 536 targets, well above the number of genes in the human genome. Spatial crowdedness could be a challenge when targeting many genes, which is common to all RCA-based approaches. With each amplicon being around 400–800 nm in diameter (45), the number of reads that can be obtained from each cell is likely limited to a couple of hundreds to lower thousands. This limitation can be overcome by combining BOLORAMIS with Expansion Microscopy (51,52), which has been successfully demonstrated by our colleagues in a recent publication (28). When compared with bulk RNA-seq data, quantifying relative gene expression levels is a limitation of BOLORAMIS. This limitation could also be overcome by performing BOLORAMIS in a hydrogel-expanded sample with Expansion Microscopy (28). The process of expanding tissue in the hydrogel removes proteins/lipids thus potentially increasing the availability of transcripts for hybridization. The physical expansion of each cell also results in spatial decrowding, giving more room for amplicons, thus producing a higher read number per cell.

SplintR Ligase has been demonstrated for detection of miRNA from extracted total RNA (15), indicating a possibility for multiplexed *in situ* detection using BOLORAMIS. Although we observed reproducible signals for miRNAs from the high-throughput singplex experiment, our attempt to detect miRNA in the multiplexed *in situ* sequencing experiment was not successful. Factors that could explain this include: (i) lower effective concentration for each probe in the multiplexed experiment (10 nM in the multiplexed experiment due to pooling of 1034 probes versus 1 μM for each probe in singplex experiment); (ii) shorter probe hybridization time in the multiplexed experiment (1 h versus 16 h); (iii) larger washing volume for multiplexed experiment (96-well plate equivalent versus 384-well plate). Optimization of RNA fixation protocol can also be helpful for miRNA detection (53). Further exploration of probe concentration, fixation method, hybridization and washing conditions might yield better miRNA detection, which is beyond the scope of this study. Although our results indicated that further optimization is needed for detection of miRNA, BOLORAMIS could bring new opportunities for highly multiplexed *in situ* detection of alternative splicing events and short transcripts, which will be useful for studying disease pathology and diagnosis (54). BOLORAMIS can also be applied to optical pooled screens (55) for detection of guide RNAs and short barcodes for perturbations. With its unique RT-free and short arm targeting strategy, BOLORAMIS can be used as a practical tool for developmental biology research, functional genomic studies, disease prognosis and novel therapeutic developments.

## DATA AVAILABILITY

Raw Illumina sequencing data for MCF7, PGP1-fibroblasts and HMC3 have been deposited to NCBI Gene Expression Omnibus and can be accessed with GSE166293. Raw Illumina sequencing data for iNGN was acquired from NCBI Gene Expression Omnibus GSE60548. BOLORAMIS probe design software is available at https://github.com/pawlowac/BoloramisProbeDesign. All data used for the figures are available in the supplementary data.

## Supplementary Material

gkab120_Supplemental_Files

## Notes

Present address: Eswar P.R. Iyer, 10X Genomics, Pleasanton, CA 94588, USA.
