# Supplementary material for: Barcoded oligonucleotides ligated on RNA amplified for multiplexed and parallel in situ analyses
Source: Nucleic Acids Res. 2021 Mar 8;49(10):e58. doi: 10.1093/nar/gkab120 (PMC8191787; doi:10.1093/nar/gkab120)
Supplement: gkab120_Supplemental_Files [file gkab120_supplemental_files.zip › BOLORAMIS_NAR_template_Supplementary_Materials_2021-02-08.pdf]

Supplementary Materials for

Barcoded oligonucleotides ligated on RNA amplified for multiplexed and parallel *in situ* analyses

Songlei Liu[1,†,*], Sukanya Punthambaker[1,2,†,*], Eswar P. R. Iyer[1,2,13,†], Thomas Ferrante[2], Daniel Goodwin[3,4], Daniel Fürth[5], Andrew C. Pawlowski[1,2], Kunal Jindal[1,2], Jenny M. Tam[1,2], Lauren Mifflin[1], Shahar Alon[3,4], Anubhav Sinha[3,4,6], Asmamaw T. Wassie[3,4,7], Fei Chen[4,8], Anne Cheng[2], Valerie Willocq[2], Katharina Meyer[1], King-Hwa Ling[1,9], Conor K. Camplisson[1,2], Richie E. Kohman[1,2], John Aach[1], Je Hyuk Lee[5], Bruce A. Yankner[1], Edward S. Boyden[3,4,7,10,11,12], George M. Church[1,2,*]

[1] Department of Genetics, Harvard Medical School, Boston, MA, 02115, USA
[2] Wyss Institute for Biologically Inspired Engineering, Harvard University, Boston, MA, 02115, USA
[3] McGovern Institute, Massachusetts Institute of Technology, Cambridge, MA, 02139, USA
[4] Media Arts and Sciences, Massachusetts Institute of Technology, Cambridge, MA, 02139, USA
[5] Cold Spring Harbor Laboratory, Cold Spring Harbor, NY, 11724, USA
[6] Harvard-MIT Health Sciences and Technology, Massachusetts Institute of Technology, Cambridge, MA, 02139, USA
[7] Department of Biological Engineering, Massachusetts Institute of Technology, Cambridge, MA, 02142, USA
[8] Broad Institute, Massachusetts Institute of Technology, Cambridge, MA, 02142, USA
[9] Department of Biomedical Sciences, Faculty of Medicine and Health Sciences, Universiti Putra Malaysia, 43400 Serdang, Selangor, Malaysia
[10] Koch Institute for Integrative Cancer Research, Massachusetts Institute of Technology, Cambridge, MA, 02142, USA
[11] Department of Brain and Cognitive Sciences, Massachusetts Institute of Technology, Cambridge, MA, 02139, USA
[12] Howard Hughes Medical Institute, Chevy Chase, MD, 20815, USA
[13] Present address: 10X Genomics, Pleasanton, CA, 94588, USA

* To whom correspondence should be addressed. George M. Church. Tel: +1 617 432 1278; Email: gchurch@genetics.med.harvard.edu
Correspondence may also be addressed to Sukanya Punthambaker. Email: sukanya@hms.harvard.edu
Correspondence may also be addressed to Songlei Liu. Email: songleiliu@g.harvard.edu

† These authors contributed equally to this work.

This PDF file includes:

Supplementary Methods

Supplementary Results

Supplementary Figure S1-S15

Supplementary Table S16

Supplementary References

This PDF file does not include:

Supplementary Table S1-S15, which is included as a separate Excel file.

SUPPLEMENTARY METHODS

TF probe design for singleplex experiment

77 TFs spanning a broad expression range (log2 FPKM 0-9) were mined from PGP1 iNGN bulk-RNAseq data[24]. A total of 192 probes targeting 77 independent mRNA (TFs) were designed with up to 5 independent probes targeting independent region per RNA. Targeting sequences with high minimal annotated cross-hybridization were mined from Affymetrix Human Primeview arrays (Affymetrix). For all mRNA probes, total targeting region was kept constant at 25 bases, and probes were designed with asymmetric hybridization arm lengths (18/7). Ligation junctions were distributed normally around the expected frequency (mean 12 probes/Ligation Junction +/- Stdev.) with the exception of junction .CC (n = 4, ~2%). All probes targeting mRNA, MIP capture sequence was kept constant at 25 nt, with an asymmetric ligation junction positioned 18 nt from the first base of 5' targeting RNA sequence. The mean melting temperature for mRNA targeting probes was 74.35 with a Stdev. of 5.27 °C (n = 192).

miRNA probe design for singleplex experiment

77 probes targeting 77 mature human miRNA sequences were designed. miRNA targets were selected, guided from previously reported miRNA expression values in human PGP1 iPSC. Mature human miRNA sequences were mined from miRbase. For miRNA with more than one mature sequences, the mature sequence with higher reported RNA seq values were selected. The mean miRNA length was 22 nt with a Stdev. of +/- 0.93. The smallest mature miRNA target sequence was 18 nt (hsa-miR-151b). Probe-ligation junction positions were determined between positions 7-15 nt from 5', with a mean position of 11 +/-2.6 nt (Stdev.). Positions were selected against G in donor or acceptor positions to increase ligation efficiency (1, 2). The mean miRNA probe melting temperature was 64.8 +/- Stdev. of 4.46 (n = 77). The mean overall miRNA targeting probe length was 57.2 nt long (+/- .93 Stdev.), with minimum and maximum lengths being 53 and 59 nts respectively.

High-content 384-well BOLORAMIS library preparation

Probes were diluted to 10 µM final concentration in hybridization buffer consisting of 6X SSC with 10% Formamide. Probes were hybridized for up to 16 hours at 37 °C either on a in a warm room, followed by a brief wash in PBS to remove excess probes. SplintR ligation mix was prepared fresh and added slowly on the samples. Ligation reaction was carried out for 2 hours at room temperature with 1X SplintR Ligase mix. SplintR ligase mix consisted of SplintR ligase at 250 nM final concentration in 1X SplintR Ligase reaction buffer. As reported earlier, we also observed a high rate of non-cellular amplicons with SplintR ligase concentrations above 1 µM (3) . RCA mix consisting of 0.6 U/µl of Phi29 Polymerase (Lucigen, 30221-2) in 1X Phi29 polymerase buffer, 0.25 mM dNTPs, in DEPC treated water was added immediately after ligation step. Cells were incubated at 37 °C for 90 minutes, or overnight.

RNA profiling, high-content imaging and Image analysis

Fully automated images were acquired from 3 positions chosen randomly from each well on ImageXpress Micro high content screening microscope (MDS Analytical Technologies) using a 40x magnification. Wells with bright fluorescent artifacts were eliminated from analysis. Images were converted from 16bit to 8bit TIFFS, and analyzed using a fully automated pipeline in CellProfiler (4–6). On average, 1410 cells were imaged per target RNA, 93% of targets imaged consisted of at least 500 cells/ target or more. For each probe, on average 669 cells were imaged, and ~96% of probes consisted of at least 250 cells or more. For individual probes, mean sample size consisted of 669 cells/ probe with ~96% of probes containing at least 250 cells. Images were converted to a Maximum Intensity Projection (MIP). Nuclei were defined by automated thresholding and size range 40-200 pixels using shape descriptors. Cell outlines were calculated using CellMask membrane stain (ThermoFisher) and using nucleus position as “seeds” objects. Cytoplasm area was calculated by subtracting the nuclear area from cell area. Puncta images were enhanced using a top-hat filter to enhance the rolling circle amplicons. Watershed segmentation was used to separate amplicons lying in close proximity. All steps were performed using CellProfiler 2.2.0. 3D rendering of images was done using IMARIS (Bitplane). Images were quality checked individually, and wells with any bright fluorescent artifacts were manually eliminated from analysis.

Probe statistics calculation

Summarized Statistics for each probe was calculated from mean single-cell BOLORAMIS expression values (Puncta Counts/Cell). To estimate dispersion of probability distribution for independent probes targeting the same RNA, COV was calculated for each RNA targets with at least 2 or more probes targeting independent regions.

Gene-gene proximity analysis

Using each amplicon as a centroid, we counted the numbers and recorded the gene identities of all other amplicons within a 5 μm radius (about 1/2-1/4 the size of a nuclei). Then for amplicons with the same identity across the entire dataset, we summed up the counts for each of the 96 mRNAs in their proximity. This analysis provided an approach to reveal pairwise spatial relationships between genes. For each gene pair, the gene-gene proximity value was normalized by the sum of their BOLORAMIS counts in order to mitigate the influence of the relative expression level. Weighted neighborhood voting analysis was performed as follows: for each punctum, the sum of the bulk RNA-seq log2 fold change (log2fc) values of all other puncta within a 5 μm radius were calculated. This analysis provided another angle to capture the spatial context of each puncta.

SUPPLEMENTARY RESULTS

Ligation junction and correlation with bulk mRNA measurement in singleplex experiment

We split our data from 192 TF targeting probes into 16 donor/acceptor ligation junction categories and found a close dependency between Pearson correlation and ligation junction composition, which ranged from -0.351 to 0.928 across all 16 categories. Consistent with previous reports, probes with a G in either donor or acceptor positions correlated poorly with bulk RNA-seq measurements (Supplementary Figure S3H). Interestingly, C in donor/acceptor positions (dT/C, dC/C, dA/C or dC/A) displayed the highest correlation with RNA-seq values (Pearson's r: 0.928, 0.856, 0.573 and 0.57 respectively) (Supplementary Figure S3H).

Hybridization arm melting temperature and correlation with bulk mRNA measurement in singleplex experiment

We also observed a dependency between probe melting temperatures (T_m) and Pearson's correlation with bulk RNA-seq values, which explained some of the variance associate with the data. In general, probes with greater T_m's displayed a higher Pearson's correlation with bulk RNA-seq values (R^2 = 0.721, Supplementary Table S14).

Spatial patterns of transcripts observed in singleplex experiment

Single-cell spatial distribution of BOLORAMIS spots seemed non-random, and consistent for a given probe. To quantify differences in spatial localization, we calculated the ratio of mean nuclear to cytoplasmic amplicon counts (N: C ratio) and identified several transcripts with signal preferentially localized in the nucleus or cytoplasm in iPSCs (Supplemental Figure S3I). For example, we observed a significantly higher nuclear signal from probes targeting SMAD1, hsa-miR-148b-3p, POU4F2, HEY1, GBX2, FOXD3, hsa-miR-301-3p, OTX2 and NFATC1 (Z-score N: C ratio >=1.64) (Supplemental Figure S3I). In contrast, probes targeting KAT7, IRX4, THAP11, ZFX, TLX3, SOX8 and LIN28 showed higher cytoplasmic localization (Z-score N: C ratio <=-1.64) (Supplemental Figure S3I).

SUPPLEMENTARY FIGURES

Supplementary Figure S1. Representative images from the hybridization arm length test. (**A**) Perfect matching probes. (**B**) Corresponding negative control probes with a single mismatch at the ligation junction.

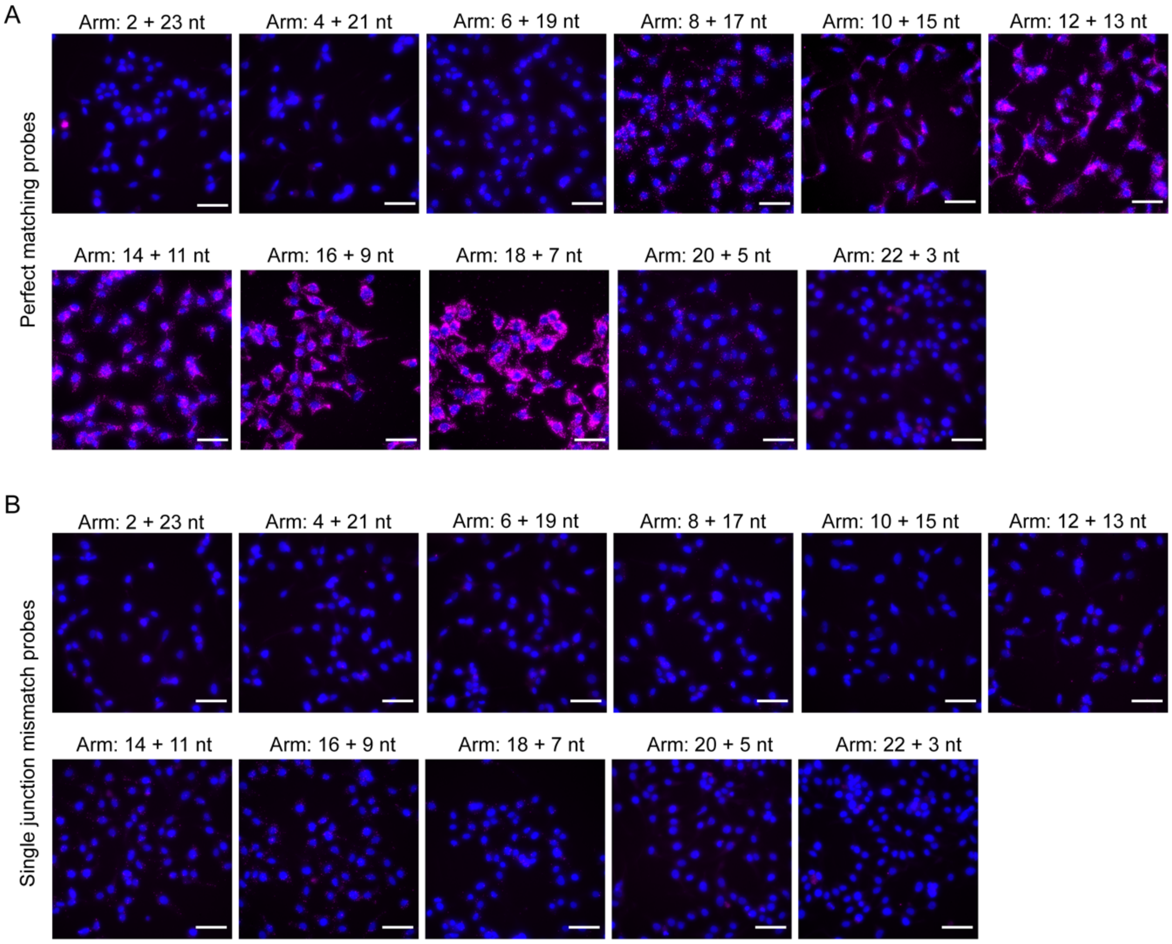

Supplementary Figure S2. High-content imaging and analysis pipeline for singleplex BOLORAMIS experiments. Cells were cultured and processed in parallel in a 384-well plate and stained. (**A**) Thousands of images representing tens of thousands of cells were collected in three optical channels using a fully automated high-content screening microscope and stored in a central database. Z-stacks were projected using Maximum-intensity projection, and images were down-sampled from 16 bit to 8-bit tiff format. (**B-K**) A custom cell-profiler pipeline was developed for segmenting and analyzing the images. Briefly, the nuclei were defined by automated thresholding and using shape descriptors (**B,E**). The cell-outlines were calculated using cell-mask membrane stain and the nucleus was used as a "seed" object for each cell (**C,F**). BOLORAMIS protocol was used to generate amplicons from targeted RNA and puncta were segmented using a top-hat filter (**D,G**). Cytoplasmic boundaries were calculated by subtracting nuclear area from total cell-boundaries (**H**). Finally, the identified puncta and other objects were computationally associated with each cell using spatial coordinates such that the spatial identity is preserved (**I,J,K**)

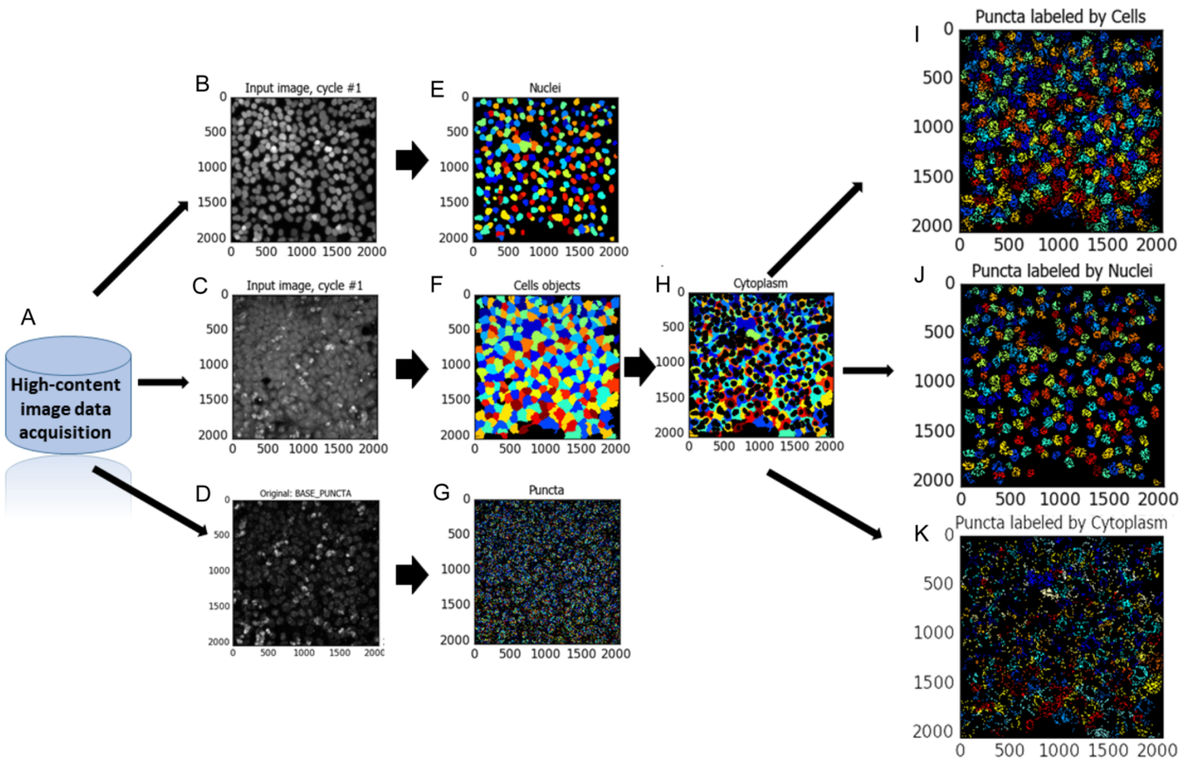

Supplementary Figure S3. Singleplex BOLORAMIS detection of coding and non-coding RNA. (**A**) Mean BOLORAMIS spot/cell measurements for 154 mRNA and miRNA measured from human iPSCs. (**B,C**) Single cell BOLORAMIS expression distribution of key coding and small-noncoding cell-type markers are shown. (**D**) Distribution of COV for independent probes targeting the same mRNA transcripts. (**E**) Mean spots per cell for tested triplicates for mRNA probes. (**F**) Mean spots per cell for tested duplicates for miRNA probes. (**G**) Correlation plot of mean single-cell BOLORAMIS values for 77 miRNAs assayed in replicates. (**H**) Effect of ligation junction composition on Pearson's correlation of mRNA measurements with bulk RNA-seq. (**I**) Scatterplot showing nuclear to cytoplasmic RNA distribution. N: C ratio values with Z scores ≥ 1.64 or ≤ -1.64 are highlighted in red and blue respectively.

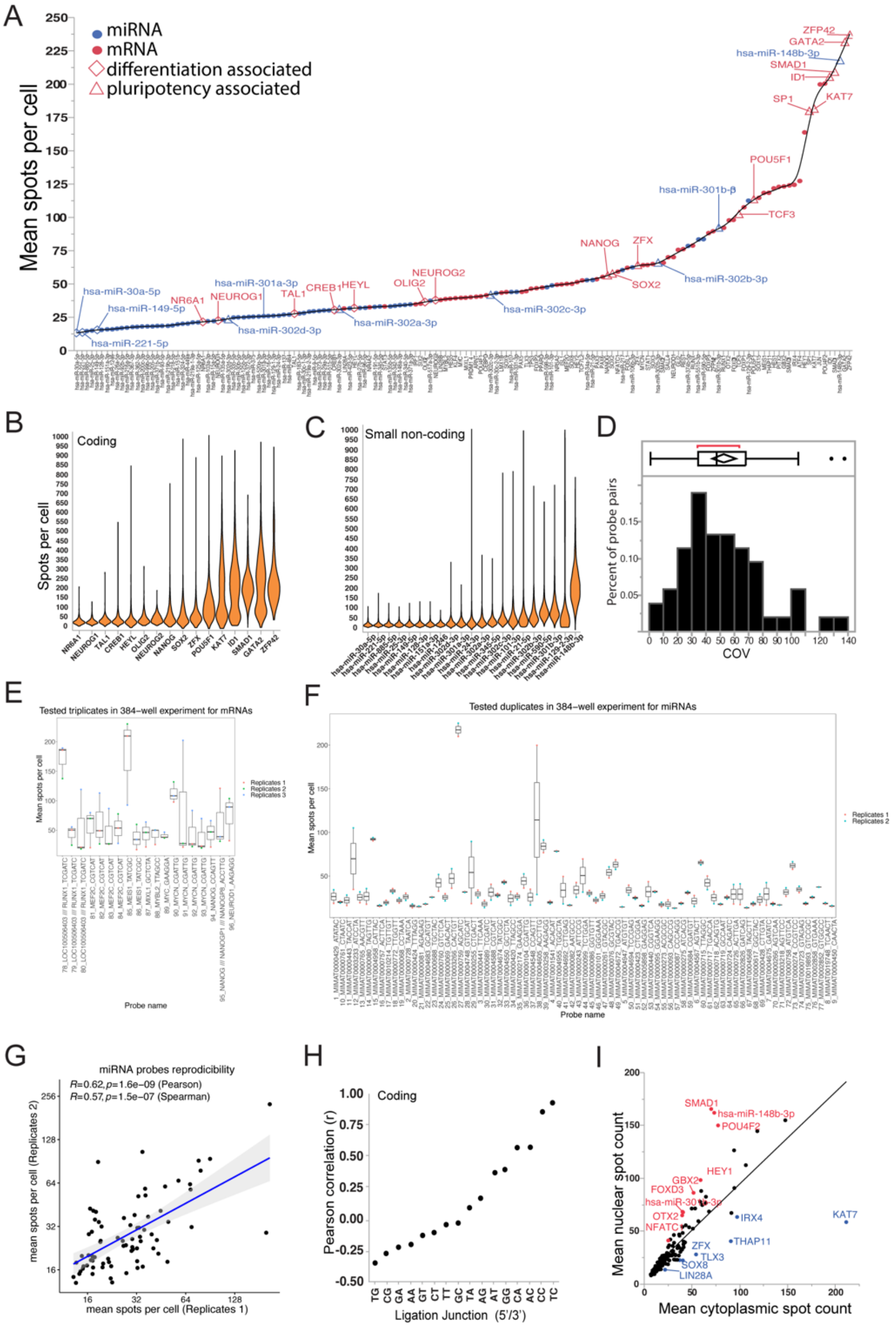

Supplementary Figure S4. Examples of BOLORAMIS phenotypes of coding and small non-coding RNAs: (**A**) GATA2, **(B**) miR-302b-3p, (**C**) SMAD1. (**D**) Sox2, CellProfiler segmented cell boundaries are indicated as red outlines. Detected spots are indicated by green diamonds for clarity. Scale bar: 50 µm.

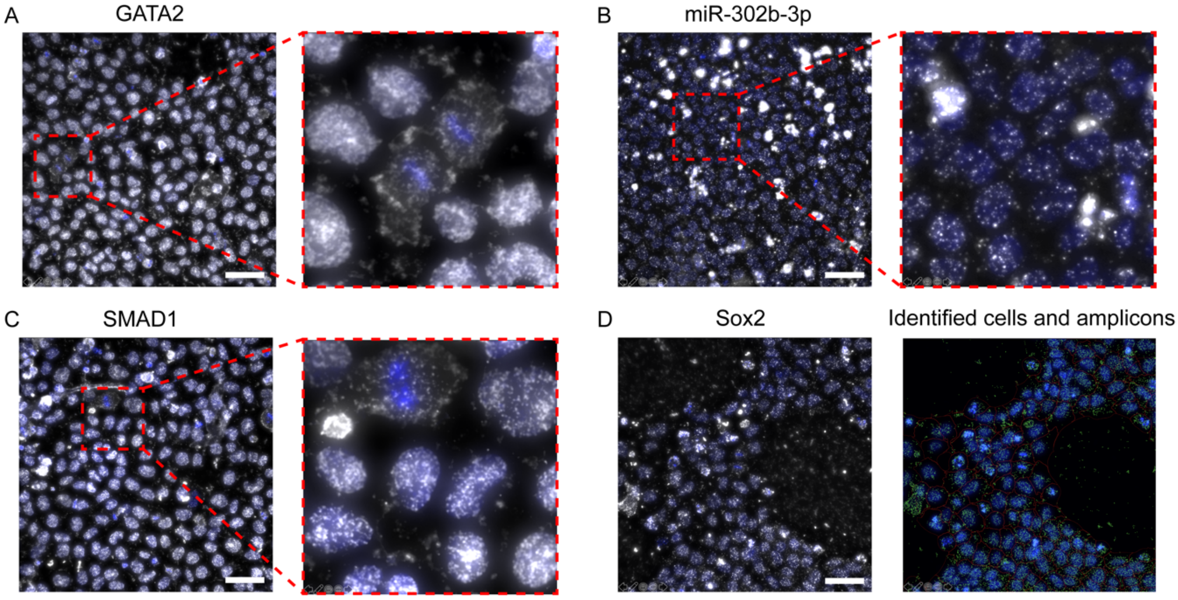

Supplementary Figure S5. Probe number per gene test. Relationship between puncta per cell and probe per gene for eight genes in (**A**) MCF7 cells and (**C**) PGP1-Fibroblasts. Correlation between BOLORAMIS detection and bulk RNA-seq (TPM) for eight genes when 2, 5, 10, or 24 probes were used for each gene in (**B**) MCF7 cells and (**D**) PGP1-Fibroblasts.

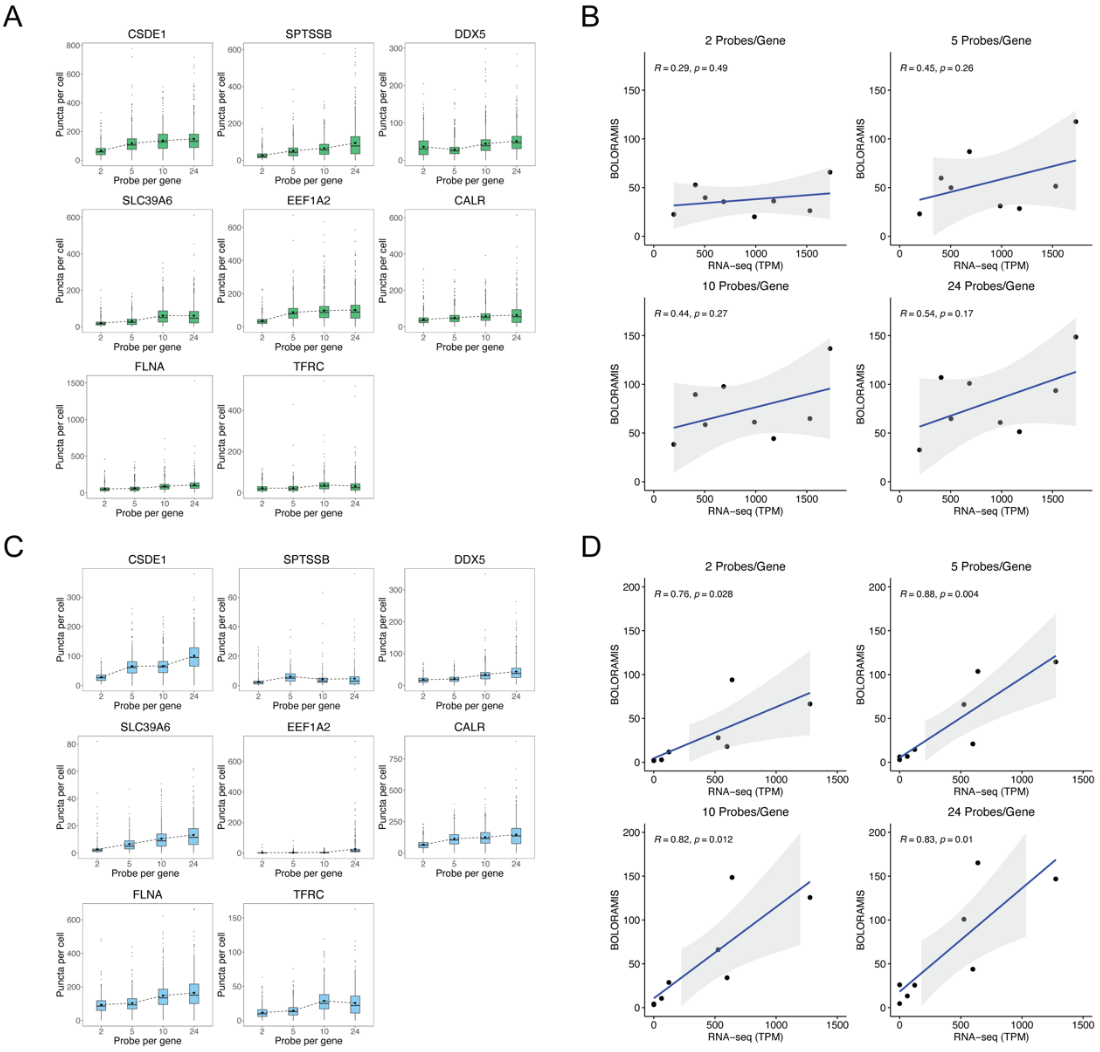

Supplementary Figure S6. Reproducibility of BOLORAMIS measurements in 96-well experiments. Eight genes were tested in duplicates in (**A**) MCF7 cells and (**B**) PGP1 fibroblasts. Two genes were tested in triplicates from (**C**) MCF7 cells and (**D**) HeLa cells.

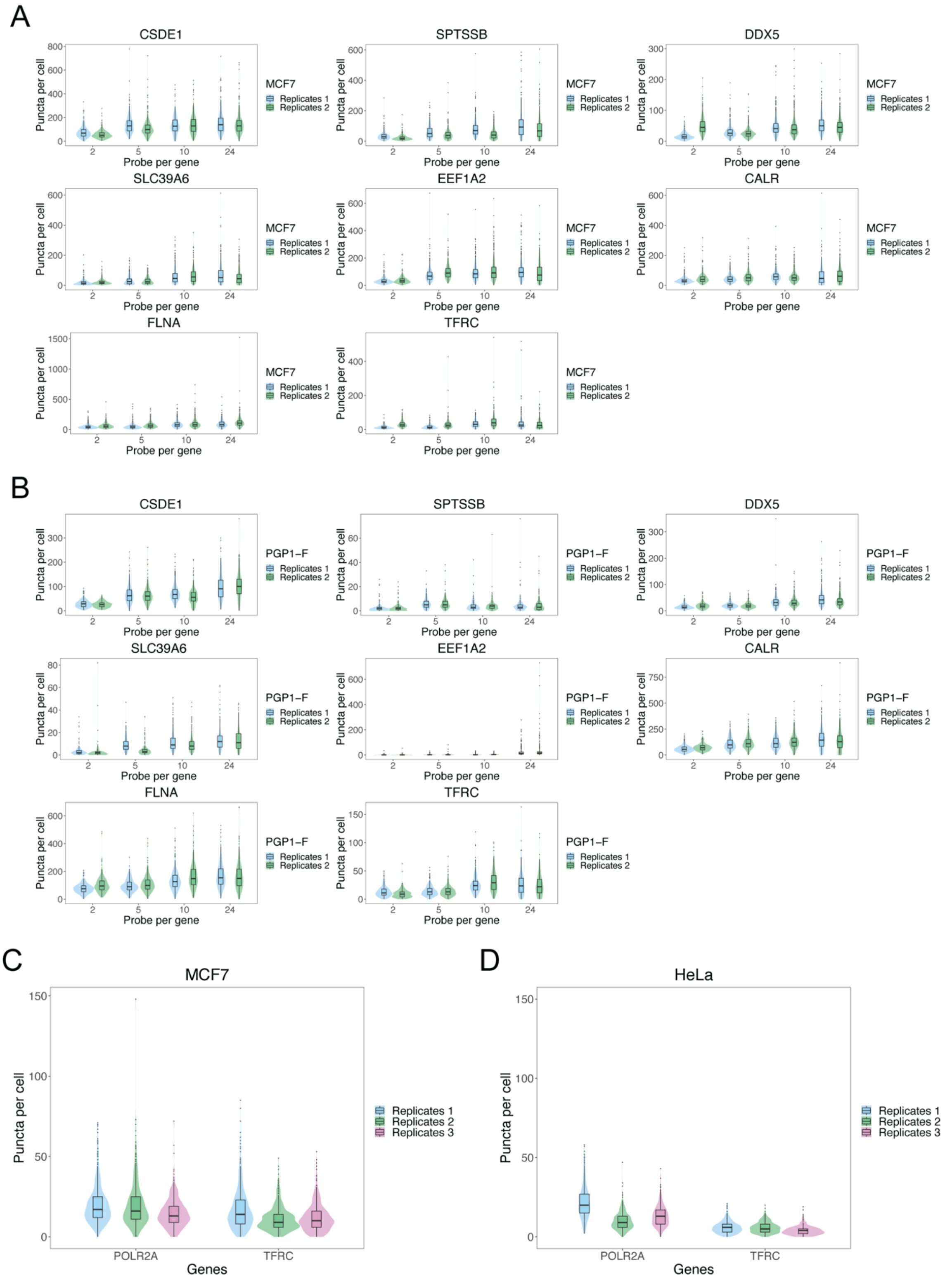

Supplementary Figure S7. Automated probe design software for multiplexed BOLORAMIS.

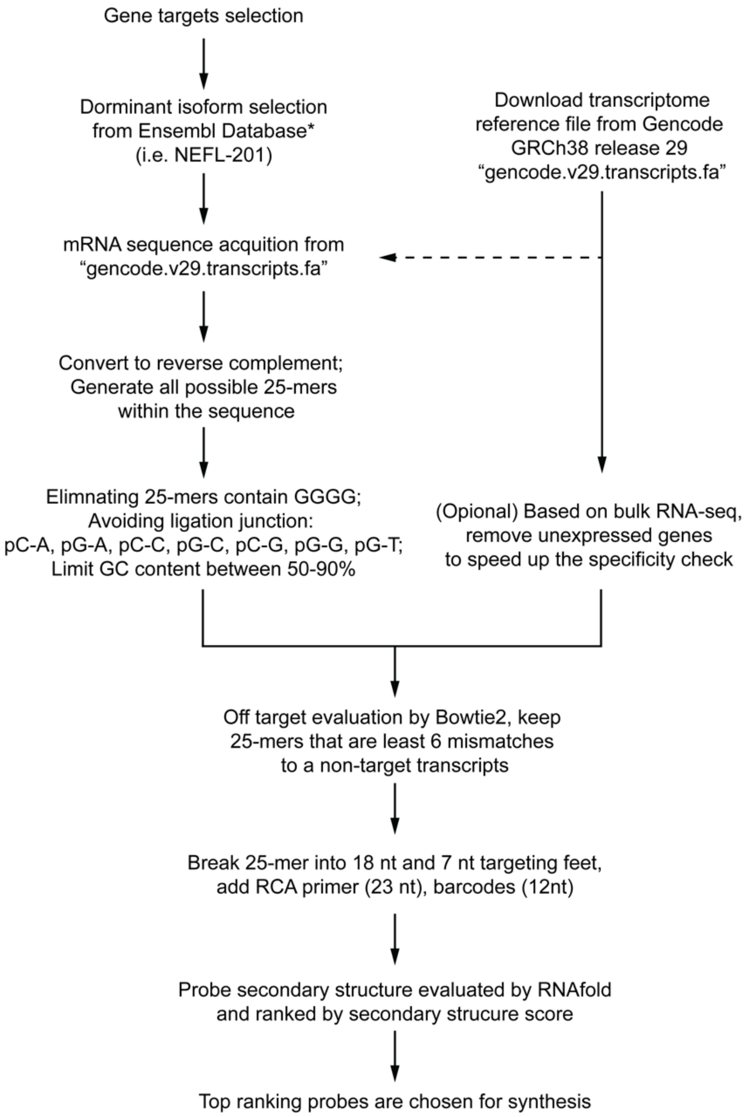

Supplementary Figure S8. Probe structure and nonamer sequencing-by-ligation. (**A**) Schematic of barcoded probe design for multiplex *in situ* sequencing. (**B**) Schematic of nonamer sequencing-by-ligation, with base #2 as an example for either minus direction or plus direction. (**C**) Eight cycles of nonamer sequencing by ligation during the multiplexing experiment (blue = G, green = A, red = C, magenta = T).

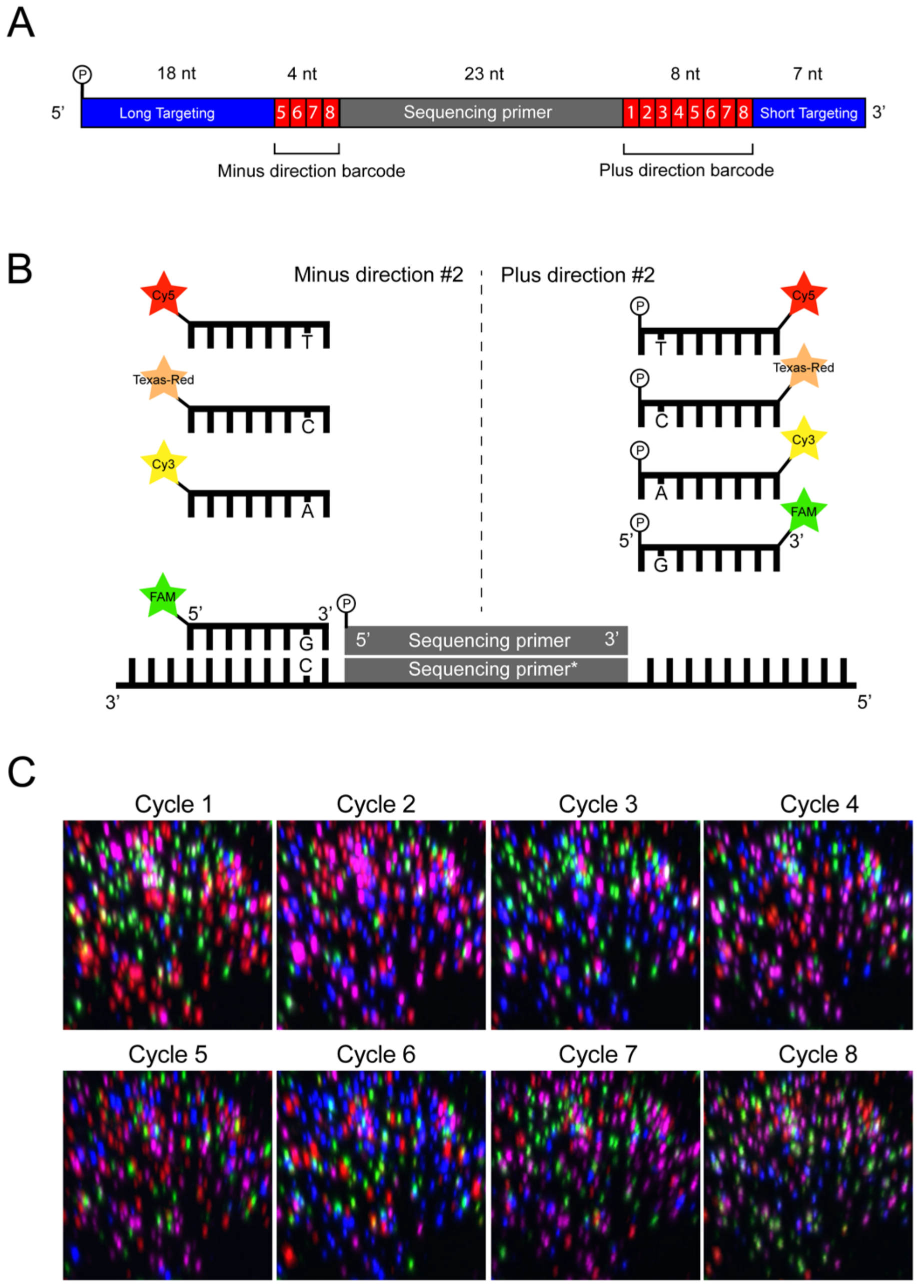

Supplementary Figure S9. Error correction of non-perfect matching barcodes. (**A**) Distribution of minimum Hamming distances to reference barcodes for all non-perfect matching barcodes. (**B**) Correlation between amplicon counts from perfect-matching and error-correctable barcodes. (**C**) Hamming distances of all 27,765 non-prefect matching barcodes to 96 mRNA (Barcode_001-096) and 77 miRNA (Barcode_097-173) reference barcodes.

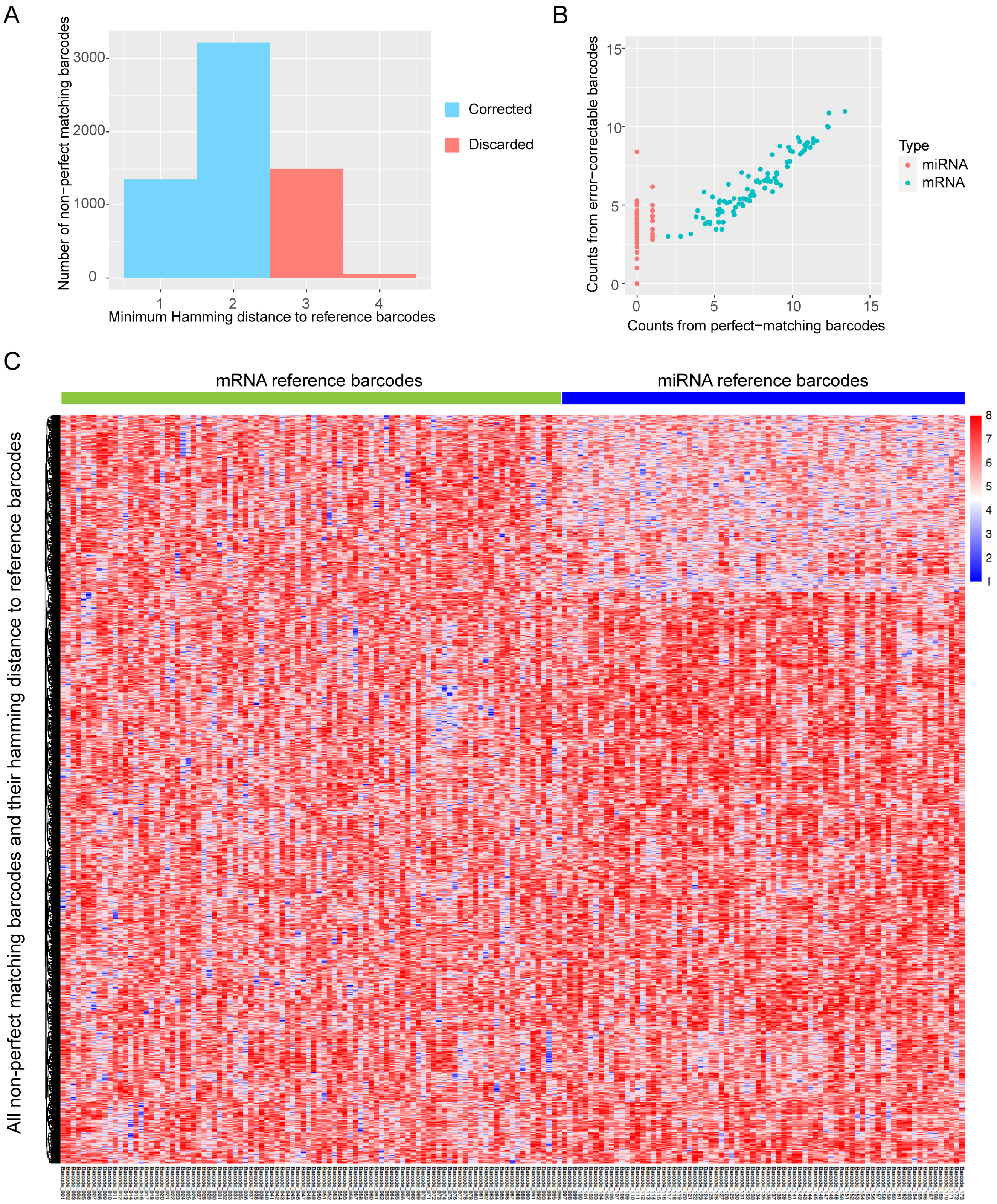

Supplementary Figure S10. Expression profile of 96 mRNAs detection by BOLORAMIS (**A**) Bar plot of BOLORAMIS count for all 96 mRNA targets. (**B,C**) Correlation between BOLORAMIS count and RNA-seq values before (B) and after (C) barcode error correction. RNA-seq values were calculated as a weighted average of HMC3 and iNGN expression value with a ratio of 4:1, according to single-cell clustering analysis.

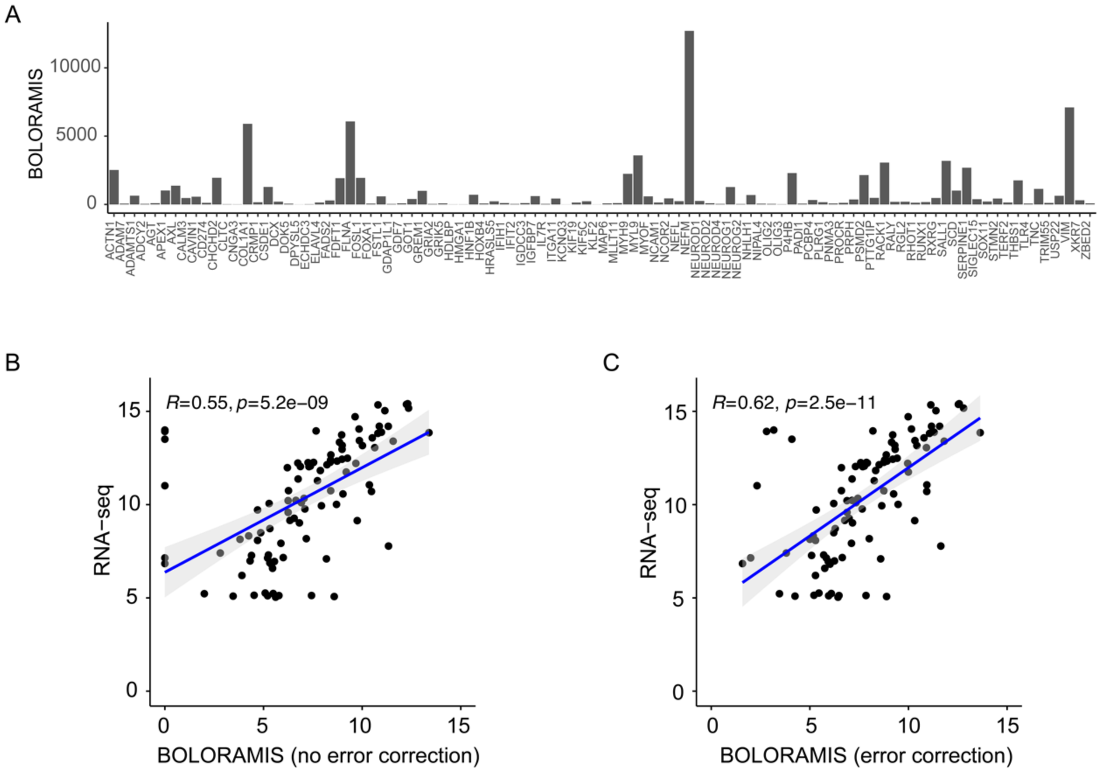

Supplementary Figure S11. Single cell segmentation. (**A**) Tile scan strategy during nonamer sequencing. Of note, even though x and y seem inverted here, it's consistent with coordinates provided in Supplementary Table S10. (**B**) Segmented single cell ROIs (black lines) and amplicons (purple dots) visualized. (**C**) Two example tiles showing relationships between cell nucleus (blue), amplicons (green), and segmented ROIs (yellow). (**D**) Centroids of single cells.

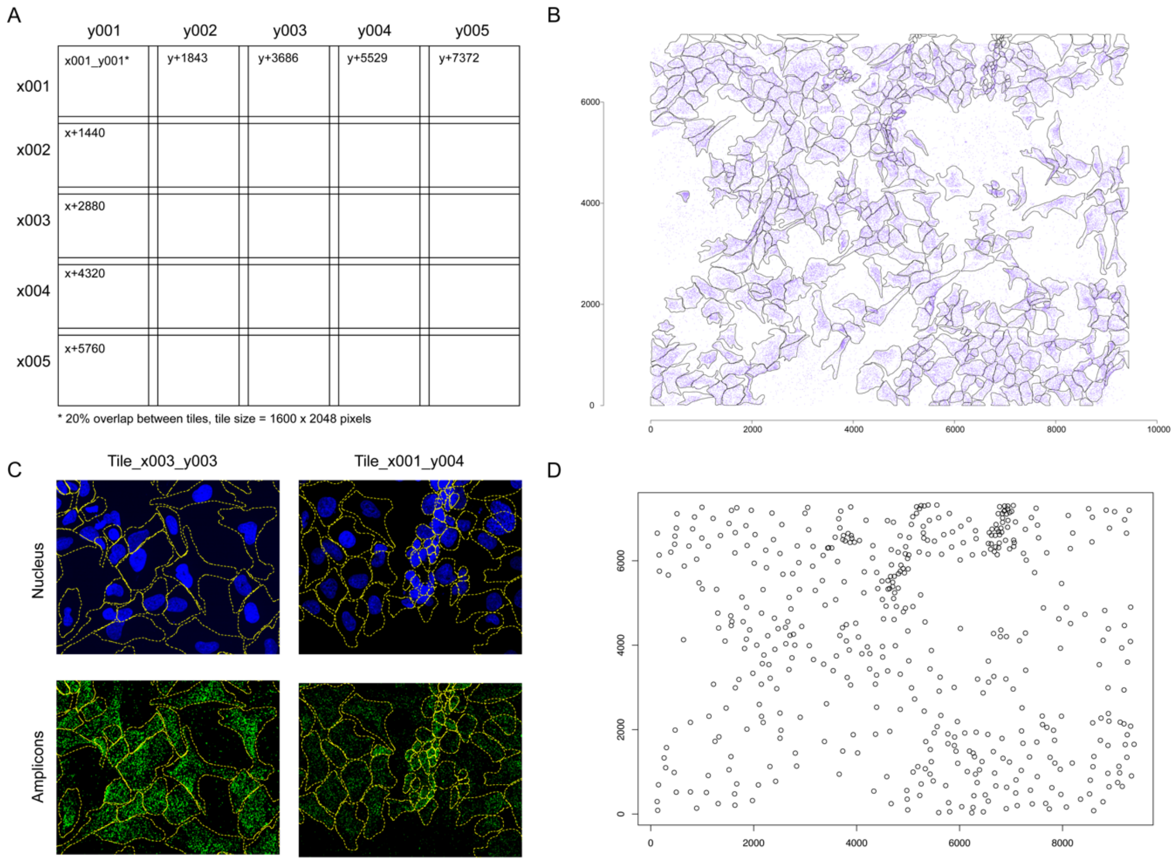

Supplementary Figure S12. Giotto QC and cluster marker distribution. (**A**) Number of genes detected per cell after QC. (**B**) Number of reads per cell after QC. (**C**) Principle component analysis. (**D**) Violin plots for iNGN and HMC3 cluster markers.

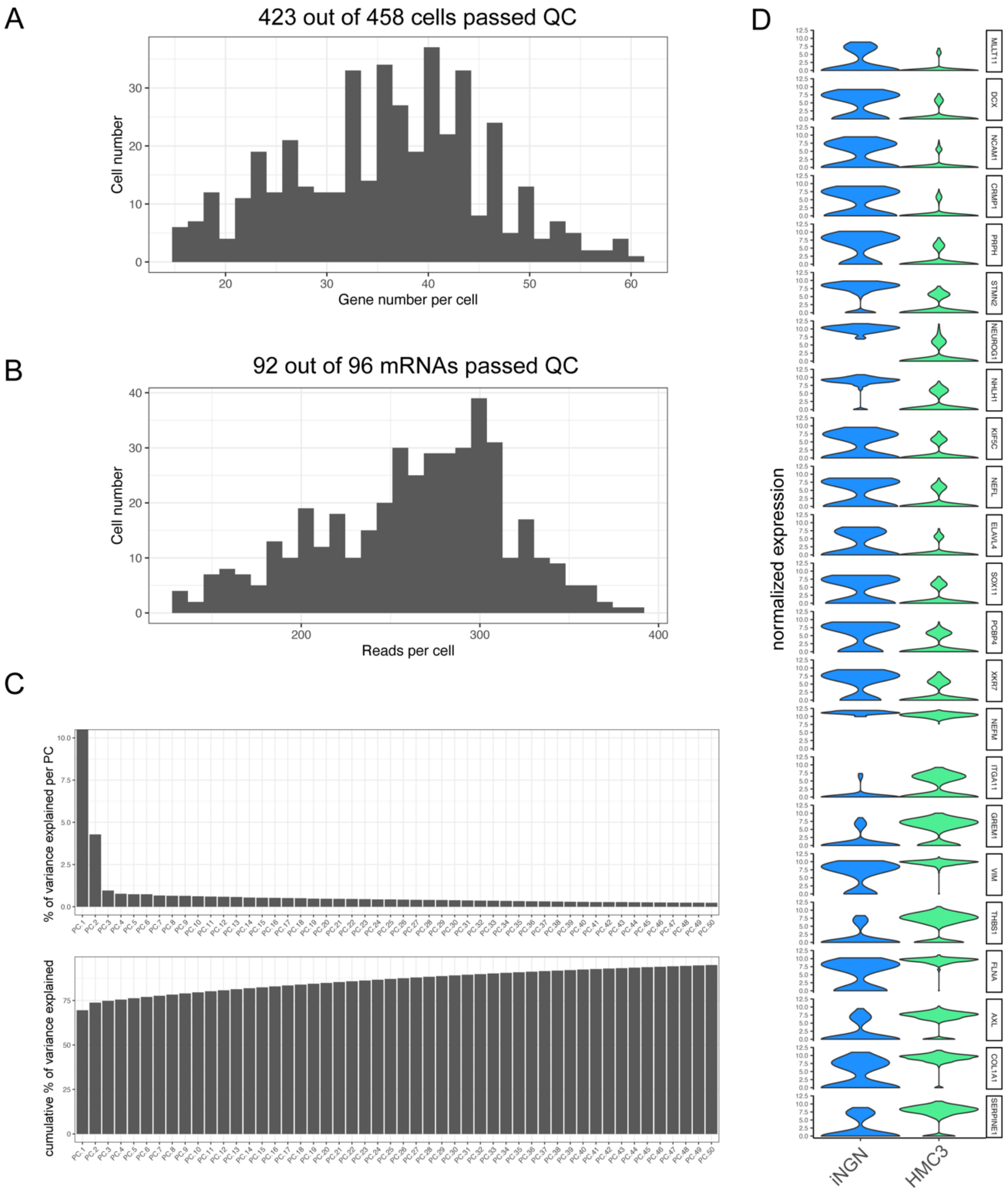

Supplementary Figure S13. Cluster and spatial distribution of additional post-QC mRNA targets. (**A**) iNGN-enriched genes, including MLLT11, DCX, NCAM1, CRMP1. (**B**) HMC3-enriched genes, including SERPINE1, COL1A1, AXL, FLNA.

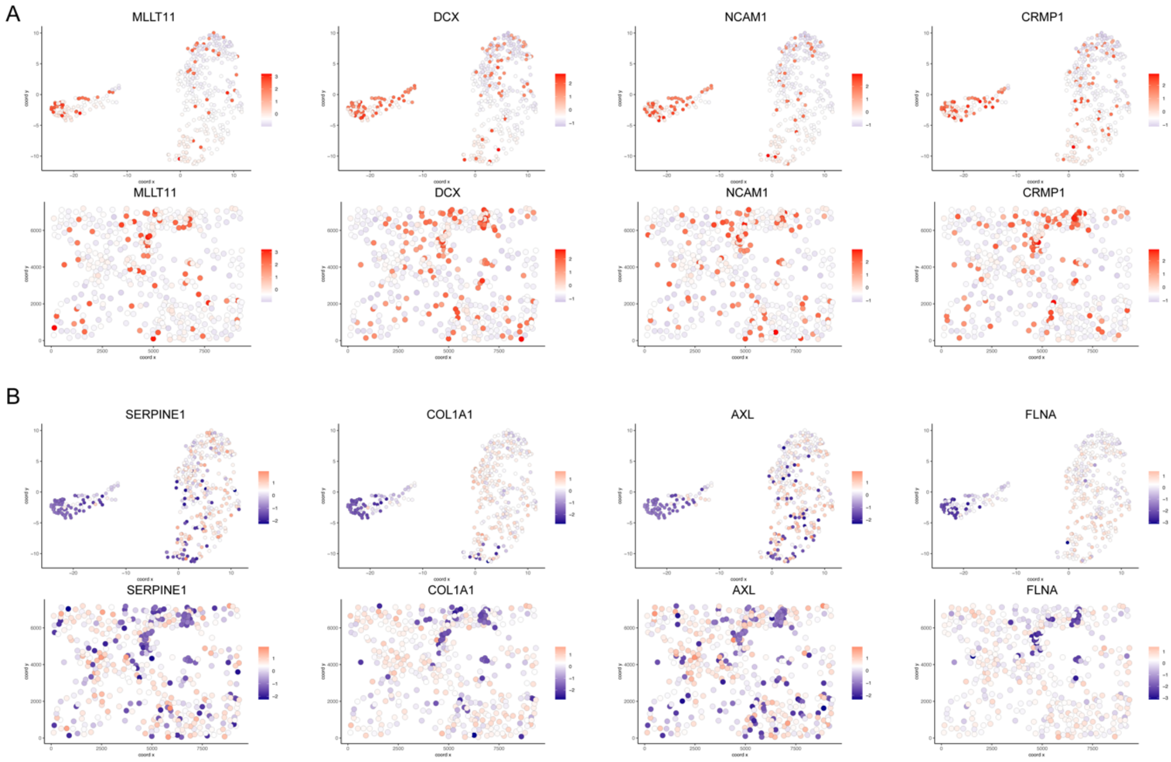

Supplementary Figure S14. Spatial clustering patterns of 96 genes captured by BOLORAMIS using gene-gene proximity analysis. (**A**) Schematic of gene-gene proximity analysis. (**B**) Correlation between BOLORAMIS counts and self-proximity value. (**C**) Weighted neighborhood voting analysis: for each punctum, the sum of the bulk RNA-seq log2 fold change (log2fc) values of all other puncta within a 5 μm radius is calculated and plotted on y axis; the log2fc value of itself (centroid puncta) is plotted on x axis. This analysis reflects the spatial context of each gene. (**D**) Heatmap for gene-gene proximity analysis for 96 genes across the entire 3D imaging volume. Automatic hierarchical clustering enabled (dist_method = “euclidean”, clust_method = “average”). Three bar graphs were appended below: top, log2 Fold Change was “log2fc” from DESeq2; middle, mean RNA-seq count was “baseMean” from DESeq2; bottom, BOLORAMIS count.

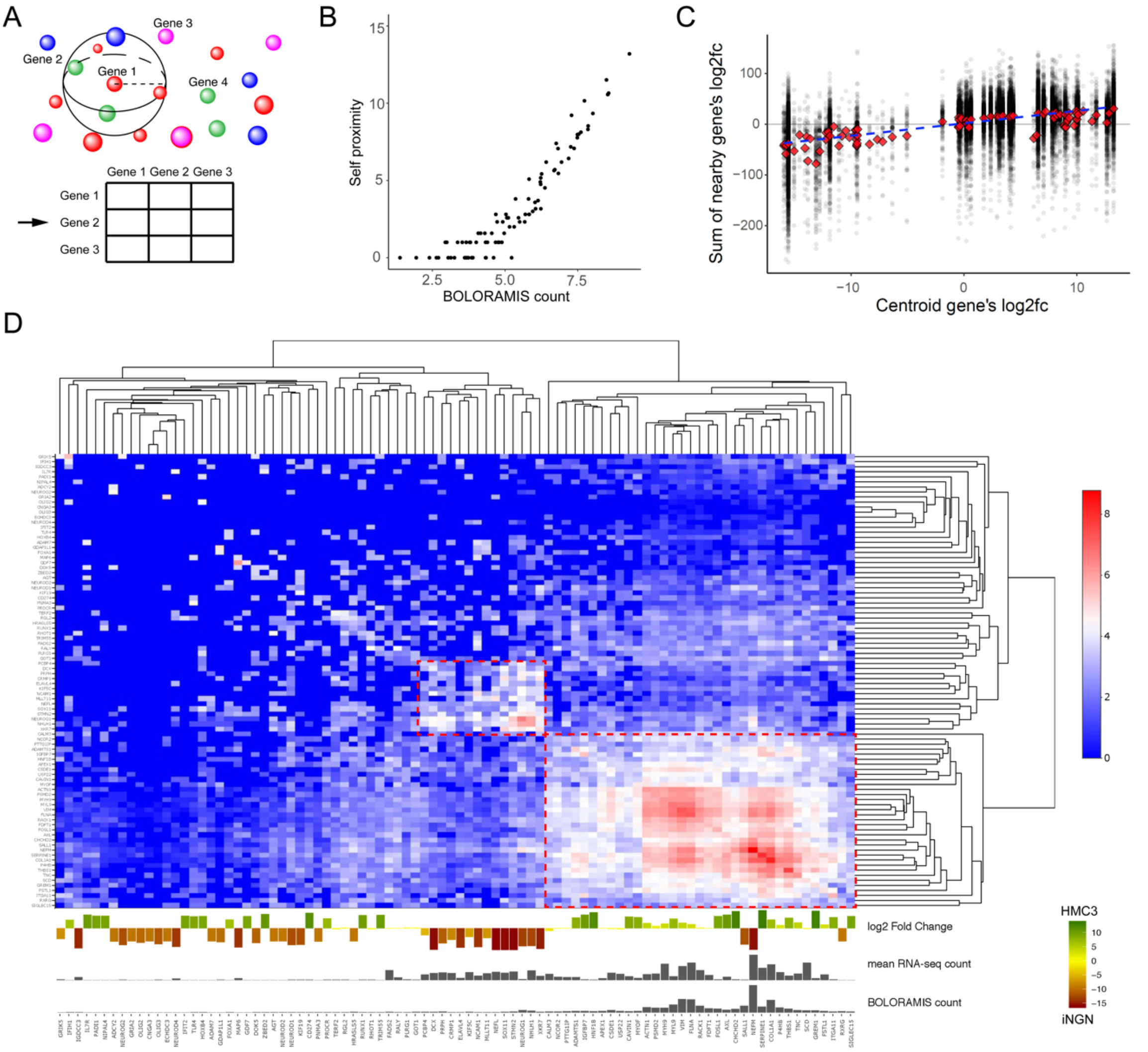

Supplementary Figure S15. Probe cost analysis of different lengths and synthesis strategy. (**A**) Comparison between cost of probe when targeting different numbers of targets for 60 nt (BOLORAMIS) and 90-100 nt (other SplintR). (**B**) Cost comparison of different synthesis strategies.

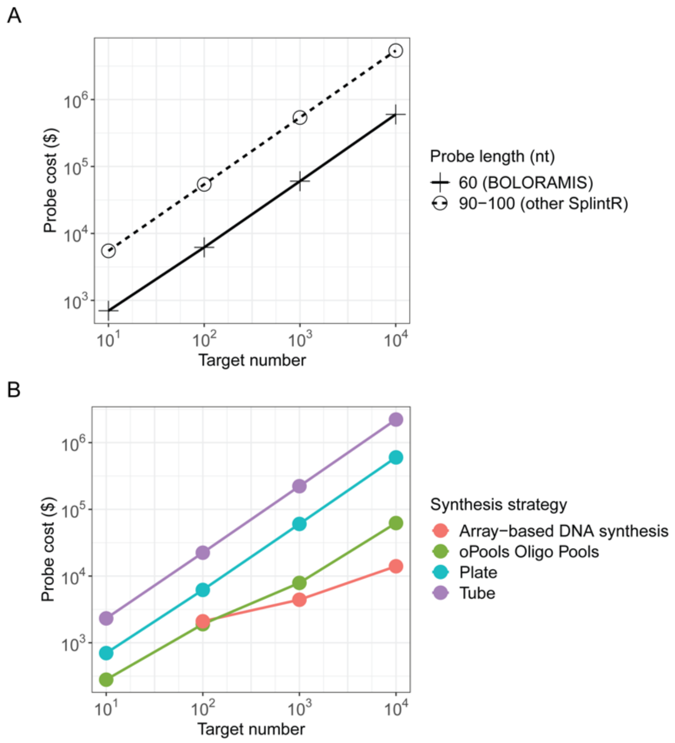

Supplementary Table S16. Comparison of targeted spatial transcriptome technologies.

Technology	Need for reverse transcription	Sensitivity	Demonstrated multiplex limits	Demonstrated subcellular resolution	Automated probe design software availability	Multiplexing chemistry	Ref.
**BOLORAMIS**	No	11-35%[1]	96 genes (957 probes)	Yes	Yes[8]	In situ nonamer sequencing by ligation	This paper
**Targeted ExSeq**	No	62%[2]	297 genes (4353 probes)	Yes	No	In situ SOLiD or Illumina chemistry	(7)
**Nilsson in situ padlock**	Yes	Not reported	99 genes (755 probes)	Yes	Yes[9]	In situ nonamer sequencing by ligation	(8, 9)
**STARmap**	No	Not reported	1020 genes	Yes	No	In situ SEDAL sequencing by ligation	(10)
**MERFISH**	No	21-105%[3]	155 genes	Yes	Yes[10]	Sequential hybridization	(11, 12)
**SeqFISH+**	No	49%[4]	10,000 genes	Yes	No	Sequential hybridization	(13)
**Spatial Transcriptomics**	Yes	6.9%[5]	All poly-A	No	Not applicable	In situ array and NGS	(14)
**High Definition ST (HDST)**	Yes	1.3%[6]	All poly-A	Yes	Not applicable	In situ array and NGS	(15)
**Slide-seq**	Yes	1%[7]	All poly-A	No	Not applicable	In situ array and NGS	(16)

[1]Three genes individually compared with smFISH in HeLa cells.
[2]Four genes individually tested in HeLa cells. Sensitivity was determined by comparing spots per cell from HCR-ExFISH and Targeted ExSeq. Two methods were performed sequentially on the same cells.
[3]Measured in U-2 OS cells. Spots per cell were compared with smFISH. 21%-94% in non-expanded samples. 105% in expanded samples, likely due to spatial decrowding of highly abundant genes.
[4]Measured in NIH/3T3 cells. Spots per cell were compared with smFISH.
[5]Measured in mouse brain tissue. ST detection events compared with spatially binned spot counts of smFISH.
[6]Measured in mouse brain tissue. Quantification methods similar to 5.
[7]Measure in mouse brain tissue. Compared spot counts with HCR-amplified smFISH.
[8] https://github.com/pawlowac/BoloramisProbeDesign
[9] https://github.com/Moldia/multi_padlock_design
[10] https://www.protocols.io/view/rna-imaging-with-merfish-design-of-oligonucleotide-menc3de

Supplementary Tables S1-S15 are included as a separate Excel file.

Supplementary Table S1. General probe sequences

Supplementary Table S2. Probe sequences for 384-well singplex experiment (192 mRNA probes, 77 miRNA probes)

Supplementary Table S3. Probe sequences for multiplexed experiment (957 mRNA probes, 77 miRNA probes)

Supplementary Table S4. Quantification of feet length experiment (Figure 1E,F)

Supplementary Table S5. Targeting region specificity analysis for human-mouse cell co-culture experiments

Supplementary Table S6. Quantification of specificity experiment (Figure 2C,D)

Supplementary Table S7. Quantification of sensitivity experiment (Figure 2G)

Supplementary Table S8. DESeq2 analysis of 96 mRNA targets in the multiplexing experiment (sorted by log2FoldChange, HMC3 vs iNGN)

Supplementary Table S9. 8-base barcodes for 96 mRNAs and 77 miRNAs in the multiplexing experiment

Supplementary Table S10. Spatial coordinates and barcode sequences of 94,175 identified amplicons

Supplementary Table S11. Gene-by-cell expression matrix for Giotto input

Supplementary Table S12. List of cell centroids for Giotto input

Supplementary Table S13. Probe cost analysis

Supplementary Table S14. Effect of probe hybridization temperature on 384-well singleplex BOLORAMIS correlation with published RNA-seq in hiPSC

Supplementary Table S15. Effect of probe ligation junction on 384-well singleplex BOLORAMIS correlation with published RNA-seq in hiPSC

SUPPLEMENTARY REFERENCES

1. Lohman,G.J.S., Zhang,Y., Zhelkovsky,A.M., Cantor,E.J. and Evans,T.C. (2014) Efficient DNA ligation in DNA-RNA hybrid helices by Chlorella virus DNA ligase. *Nucleic Acids Res.*, **42**, 1831–1844.

2. Jin,J., Vaud,S., Zhelkovsky,A.M., Posfai,J. and McReynolds,L.A. (2016) Sensitive and specific miRNA detection method using SplintR Ligase. *Nucleic Acids Res.*, **44**, e116.

3. Schneider,N. and Meier,M. (2016) Efficient In Situ Detection of mRNAs using the Chlorella virus DNA ligase for Padlock Probe Ligation. *RNA*, 10.1261/rna.057836.116.

4. Carpenter,A.E., Jones,T.R., Lamprecht,M.R., Clarke,C., Kang,I.H., Friman,O., Guertin,D.A., Chang,J.H., Lindquist,R.A., Moffat,J., *et al.* (2006) CellProfiler: image analysis software for identifying and quantifying cell phenotypes. *Genome Biol.*, **7**, R100.

5. Bray,M.-A., Vokes,M.S. and Carpenter,A.E. (2015) Using cellprofiler for automatic identification and measurement of biological objects in images. *Curr. Protoc. Mol. Biol.*, **109**, 14.17.1-13.

6. Bray,M.-A. and Carpenter,A.E. (2015) CellProfiler Tracer: exploring and validating high-throughput, time-lapse microscopy image data. *BMC Bioinformatics*, **16**, 368.

7. Alon,S., Goodwin,D., Sinha,A., Wassie,A., Chen,F., Daugharthy,E., Bando,Y., Kajita,A., Xue,A., Marrett,K., *et al.* (2020) Expansion sequencing: spatially precise in situ transcriptomics in intact biological systems. *BioRxiv*, 10.1101/2020.05.13.094268.

8. Ke,R., Mignardi,M., Pacureanu,A., Svedlund,J., Botling,J., Wählby,C. and Nilsson,M. (2013) In situ sequencing for RNA analysis in preserved tissue and cells. *Nat. Methods*, **10**, 857–860.

9. Qian,X., Harris,K.D., Hauling,T., Nicoloutsopoulos,D., Muñoz-Manchado,A.B., Skene,N., Hjerling-Leffler,J. and Nilsson,M. (2020) Probabilistic cell typing enables fine mapping of closely related cell types in situ. *Nat. Methods*, **17**, 101–106.

10. Wang,X., Allen,W.E., Wright,M.A., Sylwestrak,E.L., Samusik,N., Vesuna,S., Evans,K., Liu,C., Ramakrishnan,C., Liu,J., *et al.* (2018) Three-dimensional intact-tissue sequencing of single-cell transcriptional states. *Science*, **361**.

11. Moffitt,J.R. and Bambah,D. Molecular, spatial, and functional single-cell profiling of the hypothalamic preoptic region. *Mukku*.

12. Wang,G., Moffitt,J.R. and Zhuang,X. (2018) Multiplexed imaging of high-density libraries of RNAs with MERFISH and expansion microscopy. *Sci. Rep.*, **8**, 4847.

13. Eng,C.-H.L., Lawson,M., Zhu,Q., Dries,R., Koulena,N., Takei,Y., Yun,J., Cronin,C., Karp,C., Yuan,G.-C., *et al.* (2019) Transcriptome-scale super-resolved imaging in tissues by RNA seqFISH. *Nature*, **568**, 235–239.

14. Ståhl,P.L., Salmén,F., Vickovic,S., Lundmark,A., Navarro,J.F., Magnusson,J., Giacomello,S., Asp,M., Westholm,J.O., Huss,M., *et al.* (2016) Visualization and analysis of gene expression in tissue sections by spatial transcriptomics. *Science*, **353**, 78–82.

15. Vickovic,S., Eraslan,G., Salmén,F., Klughammer,J., Stenbeck,L., Schapiro,D., Äijö,T., Bonneau,R., Bergenstråhle,L., Navarro,J.F., *et al.* (2019) High-definition spatial transcriptomics for in situ tissue profiling. *Nat. Methods*, **16**, 987–990.

16. Rodriques,S.G., Stickels,R.R., Goeva,A., Martin,C.A., Murray,E., Vanderburg,C.R., Welch,J.,

Chen,L.M., Chen,F. and Macosko,E.Z. (2019) Slide-seq: A scalable technology for measuring genome-wide expression at high spatial resolution. *Science*, **363**, 1463–1467.
